# Trade‐offs between temporal stability and level of forest ecosystem services provisioning under climate change

**DOI:** 10.1002/eap.1785

**Published:** 2018-09-04

**Authors:** Katharina Albrich, Werner Rammer, Dominik Thom, Rupert Seidl

**Affiliations:** ^1^ Institute of Silviculture University of Natural Resources and Life Sciences (BOKU) Vienna Peter Jordan Straße 82 1190 Wien Austria; ^2^ Present address: Rubenstein School of Environment and Natural Resources University of Vermont 308i Aiken Center Burlington Vermont 05405 USA

**Keywords:** biodiversity, climatic change, ecosystem services, forest management, iLand, natural disturbances, temporal stability

## Abstract

The ability of forests to continuously provide ecosystem services (ES) is threatened by rapid changes in climate and disturbance regimes. Consequently, these changes present a considerable challenge for forest managers. Management of forests often focuses on maximizing the level of ES provisioning over extended time frames (i.e., rotation periods of more than 100 yr). However, temporal stability is also crucial for many ES, for example, in the context of a steady provisioning of resources to the industry, or the protection of human infrastructure against natural hazards. How temporal stability and the level of ES provisioning are related is of increasing interest, particularly since changing climate and disturbance regimes amplify temporal variability in forest ecosystems. In this simulation study, we investigated whether forest management can simultaneously achieve high levels and temporal stability of ES provisioning. Specifically, we quantified (1) trade‐offs between ES stability and level of ES provisioning, and (2) the effect of tree species diversity on ES stability. Simulating a wide range of future climate scenarios and management strategies, we found a negative relationship between temporal stability and level of ES provisioning for timber production, carbon cycling, and site protection in a landscape in the Austrian Alps. Tree species diversity had a predominantly positive effect on ES stability. We conclude that attempts to maximize the level of ES provisioning may increase its temporal variability, and thus threaten the continuity of ES supply. Consequently, considerations of stability need to be more explicitly included in forest management planning under increasingly variable future conditions.

## Introduction

Forest ecosystems contribute to human wellbeing by providing a wide variety of ecosystem services (ES). These services range from providing wood products and drinking water to regulating services (e.g., flood protection, climate regulation), cultural services (e.g., recreation, spiritual services), and supporting services (de Groot et al. 2002, MEA 2005). Forest ecosystems also harbor high levels of biodiversity, which, in addition to its intrinsic value, is crucial for ecosystem functioning and resilience (Thompson et al. 2009).

Ongoing changes in climate and disturbance regimes raise concerns regarding the future ability of forests to provide ES to society. Many projections for the coming decades and centuries suggest negative impacts on the provisioning of ecosystem services (Breshears et al. 2011, Elkin et al. 2013, Lindner et al. 2014). Consequently, these changes introduce considerable uncertainty into the management for ecosystem services (MEA 2005). Forests, characterized by long‐lived trees with a large ecological amplitude, respond only slowly to transient changes in the climate system (Thom et al. 2017a), a fact that can contribute to the stability of forest ecosystem services provisioning in the short term. However, processes such as disturbance (i.e., large pulses of tree mortality from agents such as storms and insect outbreaks) can also rapidly change forest ecosystems (Lucier et al. 2009), with immediate and severe consequences for ecosystem services (Breshears et al. 2011). Forest disturbances are expected to increase under climate change (Schelhaas et al. 2003, Seidl et al. 2017), and have predominately negative impacts on the provisioning of ecosystem services (Thom and Seidl 2016).

Forest management has a long tradition of dealing with uncertainty, which emanates from the extensive time horizons inherent in forestry (von Detten and Hanewinkel 2017). Historically, environmental conditions were, however, considered to be stable, neglecting environmental change. Furthermore, a focus on maximizing a single ecosystem service (usually timber production) greatly simplified traditional forest management planning. Today, it is of increasing importance to take the uncertainties introduced by changes in climate and disturbance regimes into account in order to safeguard ES supply, as well as to consider a range of multiple ecosystem services simultaneously (Millar et al. 2007, Daniel et al. 2017).

Accounting for potential future changes in forest ecosystem services is challenging due to the multiple simultaneous effects that forests have on human wellbeing. One example is the question of how forests can best contribute to climate regulation and the mitigation of climate change. On the one hand, enhancing carbon (C) sequestration and storage in forests via increasing forest area and standing volume mitigates anthropogenic climate change (Canadell and Raupach 2008, Vass and Elofsson 2016). On the other hand, increasing the use of renewable resources and substituting fossil resources has benefits for the climate system, but requires increased harvesting of forest biomass (Lundmark et al. 2014), in turn reducing in situ C storage. When assessing the effect of future forest development trajectories on ecosystem services, it is thus important to consider changes in both state variables (stocks) and exchange rates of the ecosystem with its surrounding (flows).

Given the uncertainties regarding future environmental conditions, a key challenge for forest management is to foster forest development trajectories that ensure high levels of ES provisioning under a wide variety of future conditions. Planning considerations usually extend over one or several rotation periods (i.e., several decades to centuries) and thus address the long‐term provisioning of ES. However, for many ecosystem services, temporal continuity is equally important as the level of provisioning over an extensive planning period. For instance, the protection of soil from erosion has to be maintained constantly, as a single heavy rain event affecting temporally unstocked soil can result in substantial soil losses that can only be recovered over centuries to millennia (Shakesby et al. 1993). Natural disturbances are a major concern in this regard, as they can distinctly change a forest's ability to provide ES within a short period of time (hours to a few years). Consequently, addressing disturbances is a key issue in ecosystem management for services where temporal stability is of high importance (Dorren et al. 2004, Vacchiano et al. 2015).

The temporal stability of ES provisioning is therefore an important area of research. In the context of a comprehensive consideration of uncertainties in ecosystem service assessments (Runting et al. 2017), it is of interest whether management strategies can be developed that simultaneously achieve high stability and a high level of ecosystem services provisioning. While many recent studies have investigated the role of forest management in providing multiple ecosystem services under climate change, as well as dealing with trade‐offs between individual services (Temperli et al. 2012, Creutzburg et al. 2017, Irauschek et al. 2017, Mina et al. 2017, Pohjanmies et al. 2017), the question of whether stability of ecosystem service provisioning comes at the expense of the level of service provisioning has, to our knowledge, not yet been investigated.

Here, we used landscape‐scale simulation modeling to investigate the relationship between temporal stability and level of ecosystem service provisioning over a wide range of possible future forest trajectories. Specifically, we studied ES stability‐level relationships for three ecosystem services (timber production, carbon cycling, and site protection) under four alternative management strategies and six future climate and disturbance scenarios over a time frame of 200 yr. Based on previous research, we expected management approaches that supply high levels of certain ecosystem services (e.g., high timber production in monocultures of productive conifers) to be less stable under future environmental conditions (Temperli et al. 2012, Felton et al. 2016). We thus hypothesized that a significant trade‐off between the stability and provisioning of ecosystem services exists, and that an increased level of ES provisioning simultaneously results in lower stability of ES provisioning. A key aim of this contribution was to test for and quantify this trade‐off for a wide range of different ES.

To further elucidate ES stability, we subsequently investigated the role of biodiversity in stabilizing ecosystem services provisioning. A strong focus of previous ES research has been on quantifying the influence of biodiversity on ES provisioning. This research has shown that biodiversity can contribute to ecological stability, and therefore to the stability of ES provisioning, for example, by buffering impacts of climate change and disturbance on ecosystem functioning (Tilman et al. 2006, Isbell et al. 2009, Thompson et al. 2009, Mori et al. 2013, Harrison et al. 2014, Morin et al. 2014, Silva Pedro et al. 2015). However, recent findings also underline that the local context and the services considered strongly modulate the relationship between biodiversity and ecosystem functions (Ratcliffe et al. 2017). Consequently, we tested the hypothesis of a generally positive impact of biodiversity on temporal ES stability, and asked whether the effect of biodiversity on stability is similar for a range of ecosystem services.

## Materials and Methods

### Study landscape

**Figure 1:**
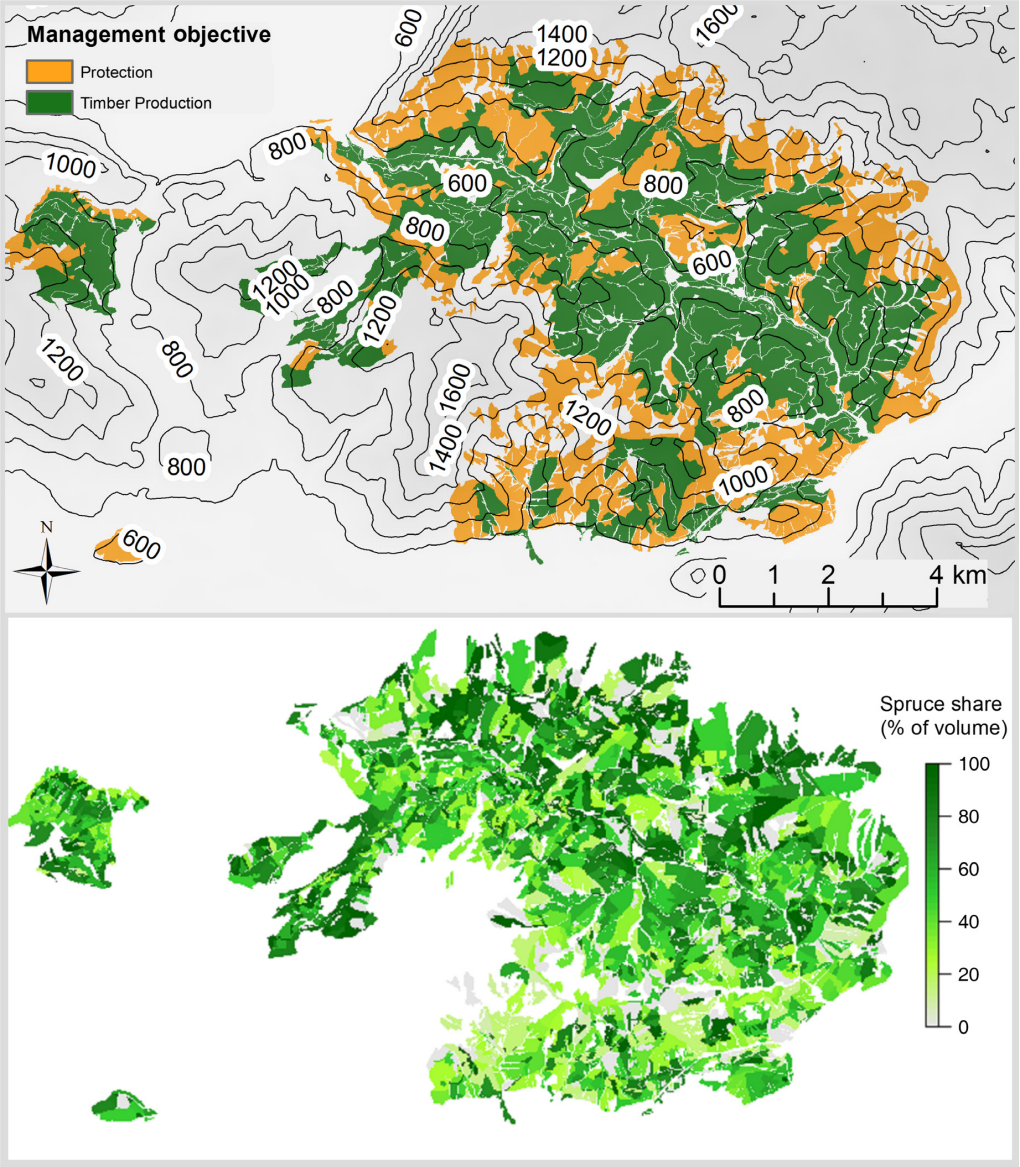
Primary management objectives under current management (upper panel) and current share of Norway spruce (lower panel) of the Weissenbachtal study landscape, Austria.

The study landscape Weissenbachtal is located in the northern front range of the Austrian Alps (47.78° N, 13.59° E), covering an area of approximately 8,100 ha in total, 5,716 ha of which are forested. The range of elevation extends from 500 to 1400 m above sea level. Climate is strongly modulated by the topographic gradients within the landscape, with temperature decreasing with elevation (from 9.6° to 5.5°C across the elevational gradient with a landscape average of 7.5°C) and precipitation increasing with elevation (precipitation range 1,207–2,071 mm with a landscape average of 1,503 mm of annual precipitation). The substrate is calcareous with predominately shallow Chromic Cambisols and Rendzic Leptosols (Matthews et al. 2017). The area is under the stewardship of the Austrian Federal Forests, who currently manage the landscape with a focus on timber production and site protection (Fig. 1). The potential natural vegetation consists of Norway spruce (*Picea abies* (L.) Karst.), European beech (*Fagus sylvatica* L.), and silver fir (*Abies alba* Mill.), with beech dominance decreasing and spruce dominance increasing with elevation (Kilian et al. 1994). The current vegetation is characterized by a strong dominance of spruce (Fig. 1), which is the result of past management focused on fuel wood production for a nearby salt mine.

### Simulation model

We applied the individual‐based forest landscape and disturbance model iLand (Seidl et al. 2012a) to investigate the effect of different management strategies and climate change scenarios on the provisioning of ecosystem services. iLand simulates processes across multiple, dynamically interacting hierarchical levels (i.e., individual tree, stand, landscape), capturing interactions between forest vegetation, environmental drivers, disturbances, and forest management. A detailed description of iLand can be found in Seidl et al. (2012a, b) and Thom et al. (2017b). Here, we focus on describing the model components of particular relevance in the context of the current study objectives. Technical model documentation as well as the executable and source code of iLand is *available online*.^1^

Tree growth is modeled based on generalized physiological principles, simulating primary production by means of a light use efficiency approach (Landsberg and Waring 1997). C allocation to tree compartments is based on allometric ratios and dynamically adapts to the abiotic and biotic environment of an individual tree. Compartment‐specific turnover rates are used to calculate the C input into four detrital pools, for which a decomposition module calculates C storage and release from the ecosystem (Seidl et al. 2012b). A process‐based water balance is calculated at a daily time step, allowing the simulation of canopy interception and evapotranspiration of water as well as snow cover and water runoff.

Process‐based disturbance modules for wind and bark beetles (specifically *Ips typographus* L., Coleoptera: Curculionidae), the two most important disturbance agents in Austria (Thom et al. 2013), were used to represent the natural disturbance regime in the simulations (Seidl et al. 2014a, Seidl and Rammer 2017). Disturbances were simulated dynamically and spatially explicitly, taking into account agent‐specific susceptibilities (such as forest structure and edge effects for wind, and host tree availability and defense for bark beetles) as well as climate conditions (peak wind speeds, temperatures influencing beetle development). Bark beetle population dynamics and spread were simulated explicitly in space and time.

To study management effects on ecosystem services provisioning and stability, we used the agent‐based forest management model ABE (Rammer and Seidl 2015), which is fully integrated into the iLand simulation framework. Based on pre‐defined targets (e.g., sustainable harvest levels), constraints for management (e.g., maximum cut‐block sizes), and information about silvicultural systems (e.g., tending and thinning regimes) ABE autonomously schedules and implements forest management operations for each stand of the simulated landscape. Furthermore, ABE dynamically tracks changes in the environment, such as disturbances or changes in tree growth, and adapts forest management accordingly (e.g., by reducing regular harvest rates to buffer the impact of disturbances), in order to ensure that an overall sustainable harvest level is met.

iLand has been successfully applied and evaluated for temperate forest landscapes in North America (Seidl et al. 2012a, b, 2014b), and Europe (Seidl et al. 2014a, Thom et al. 2017c, L. Dobor, T. Hlásny, W. Rammer, I. Barka, J. Trombik, P. Pavlenda, V. Šebeň, P. Štěpánek, R. Seidl, *unpublished manuscript*). For the Weissenbachtal landscape studied, here, we tested the model against independent data on tree growth and productivity, and evaluated the ability of iLand to reproduce the potential natural vegetation distribution across the landscape (Appendix S1: Section S2.1). The results of these tests showed that iLand was well able to reproduce expected forest dynamics at Weissenbachtal. In addition, the implementation of the management strategies (Appendix S1, Section 1.4) was tested extensively, focusing on the ability of ABE to meet prescribed management targets (see Appendix S1: Section S2.1.3 for details). Moreover, we compared the simulated carbon and water cycle to independent reference data for the region (Appendix S1, Sections S2.2, and S2.3).

### Initial conditions and climate scenarios

We used current management plans as well as forest inventory data and airborne laser scanning (≤1 m horizontal resolution) to determine stand (e.g., growing stock, species shares, horizontal structure) and tree characteristics (e.g., diameter at breast height, tree height) of the current vegetation. Soil and climate data were considered at 100 × 100 m horizontal resolution in the simulation. Soil‐related input for the simulation (effective soil depth, sand, silt, and clay content, plant‐available nitrogen, and initial soil carbon stock) were derived from the site classification system of the Austrian Federal Forests (Weinfurter 2004) in combination with quantitative soil profile data from the Austrian Forest Soil Survey (Seidl et al. 2009).

To study a wide range of potential future environmental conditions a total of six climate scenarios were analyzed, combining three climate trajectories derived from climate models with two future scenarios of peak wind speed. The three climate change scenarios were based on different combinations of global and regional circulation models under A1B forcing, namely CNRM‐RM4.5 driven by ARPEGE (Radu et al. 2008), MPI‐REMO (Jacob 2001), and ICTP‐RegCM3, both driven by ECHAM5 (Pal et al. 2007). Previous analyses showed that the temperature and precipitation changes resulting from the A1B scenario storyline lie between those expected from RCP4.5 and RCP6.0 for our study area (Thom et al. 2017a). For mean annual temperature, the three scenarios project an increase of +3.2° to +3.3°C for the period 2080–2100 relative to the climate of 1950–2000. For the same reference period, annual precipitation sum changes by between −84 and +160 mm in the studied climate scenarios. Climate was assumed to stabilize at the level of 2080–2100 for the years simulated past 2100. Climate scenario data were statistically downscaled using climate observations provided by the Central Institute of Meteorology and Geodynamics Vienna (cf. Thom et al. 2017b). To address the considerable remaining uncertainties regarding local future wind climate (Lindner et al. 2014), we studied two wind scenarios for each of the three model‐derived climate trajectories. The first scenario was based on historically observed wind data, assuming that future peak wind speeds and return intervals resembled those of the recent past. The second scenario follows recent findings indicating that future peak wind speed could increase moderately in Central Europe (Rockel and Woth 2007), assuming a 10% increase in peak wind speeds across all wind events. An in‐depth description of the disturbance scenarios and processes can be found in Seidl et al. (2018a).

### Management strategies

We investigated four alternative management strategies covering a broad range of potential future silvicultural pathways (Table 1). Two strategies were derived from a stakeholder process involving both the current managers of the landscape (Austrian Federal Forests, BAU strategy) and the local forest authorities tasked with supervising forest management in the area (Forest Service Upper Austria, AM1 strategy). The BAU strategy represents current business as usual management, featuring a strong emphasis on the tree species Norway spruce and European larch (*Larix decidua* L.), with European beech as admixed species. Rotation periods were between 120 and 140 yr (increasing with elevation), and one to two thinning interventions were conducted in the first half of the rotation period. In contrast, the AM1 strategy also includes silver fir and Scots pine (*Pinus sylvestris* L.) as important target tree species. Rotation ages were reduced to 120 yr also in higher elevation stands, and two thinnings were conducted in all stands. The AM1 strategy thus reflects the frequent recommendation to shorten rotation periods and increase thinning frequency in order to adapt forest management to climate change (Seidl et al. 2011, Loisel 2014).

**Table 1:** Overview of the four management strategies simulated

Management strategy	Description	Primary aim	Target tree species
HIST	historical management	maximum timber yield	*Picea abies*
BAU	current management by local foresters	sustainable timber production and site protection	*Picea abies*,* Larix decidua*,* Fagus sylvatica*
AM1	recommendations of local forest authority	site protection and sustainable timber production, adaptation to climate change	*Picea abies*,* Larix decidua*,* Fagus sylvatica*,* Abies alba*,* Pinus sylvestris*
AM2	based on future potential natural vegetation	provisioning of multiple forest ecosystem services under climate change	*Picea abies*,* Fagus sylvatica*,* Abies alba*,* Pinus sylvestris*,* Quercus robur*,* Quercus petraea*

In addition to these two strategies developed with stakeholders, two bracketing management strategies were designed (HIST and AM2), representing contrasting alternative management options. The HIST strategy is based on historical maximum yield management and has Norway spruce as its sole target tree species. While spruce was the only species that was actively planted and favored in thinnings, we also allowed other species to regenerate naturally under the HIST strategy. Rotation periods and thinning regimes were the same as for the BAU strategy. At the other end of the spectrum, the AM2 strategy represents an active adaptation of the tree species composition to future climatic conditions, based on the future potential natural vegetation. In order to obtain a quantitative estimate of the latter, we ran unmanaged iLand simulations from bare ground for 1,500 yr under the climate change conditions projected for the period 2080–2099. The thus derived target tree species composition of the AM2 strategy consisted of a diverse set of tree species, including species promoted in other strategies (spruce, fir, beech, pine), but also warm‐adapted native tree species such as oaks (*Quercus petraea* Matt., *Quercus robur* L.). Rotation periods and thinning regimes under AM2 followed the AM1 strategy.

All strategies were implemented in the simulations at the level of stands (average stand size 3.4 ha, with a total of 1,678 stands on the landscape) using stand treatment programs (stp), which prescribe target species shares and silvicultural treatments taking into account site conditions and the elevation of each stand (see Appendix S1: Section S1.4 for details). This resulted in a considerable within‐landscape variation in management even within a management strategy (cf. Fig. S26). The scheduling of treatments in the simulation was dynamically done by ABE. In accordance with the Austrian forest act (Anonymous 2017) disturbed areas were salvage harvested in all management strategies in order to mitigate the spread of bark beetles into neighboring stands. In total, 24 unique combinations of management strategies (four) and climate scenarios (six) were simulated, all starting from current forest conditions. In order to account for the stochasticity in the simulations (e.g., with regard to the initial location of outbreak spots of bark beetle disturbances), we simulated 20 replicates of each combination of climate scenario and management strategy (see Appendix S1: Table S4 for an analysis of variance among replicates), resulting in a total of 480 model runs. Each simulation was run for 200 yr.

### Temporal stability and level of provisioning of ecosystem services

We focused our analysis on three ecosystem services, which are of high relevance both locally and at the global scale, namely timber production, carbon cycling, and site protection. For each ES, we investigated stock and flow indicators in order to comprehensively assess the level and stability of ES provisioning under changing climate and disturbance regimes. Timber production is currently the main objective of the Austrian Federal Forests in managing the landscape. It is expected to remain highly relevant also in the future, in order to provide society with locally sourced, renewable resources. We chose standing timber volume (m^3^/ha) and annual harvest level (m^3^·ha^−1^·yr^−1^, including both planned harvest and salvage harvest after disturbances) as stock and flow indicators, respectively.

The role of forest ecosystems in carbon cycling and the mitigation of climate change through carbon sequestration and storage has received increasing attention in recent years (Fahey et al. 2010, Thom et al. 2017b). We therefore chose two indicators quantifying forest carbon cycling, Total Ecosystem Carbon (TEC, i.e., the sum of all ecosystem carbon pools simulated in the model, including carbon in stems, branches, foliage, coarse and fine roots, regeneration, snags, downed woody debris, as well as in litter and soil organic matter, in Mg C/ha) and net ecosystem productivity (NEP, i.e., the annual C uptake or release of the forest landscape, in Mg C·ha^−1^·yr^−1^). Positive NEP denotes a net uptake of carbon from the atmosphere, and thus a mitigating effect on climate change.

Site protection against soil erosion from heavy rain events and gravitational forces is of particular importance in our study landscape, given its steep mountainous terrain and shallow soils (Reger et al. 2015). As site protection is closely related to the presence of a dense forest cover (Borrelli et al. 2017), we chose Leaf Area Index (LAI, in m^2^/m^2^) as the state indicator for site protection. Furthermore, since water is a main driver of erosion we used annual water runoff (i.e., the excess water that cannot be stored in the soil or taken up by plants during the year, in mm/yr) as flow indicators of site protection against erosion. A high level of site protection is indicated by high water retention of the ecosystem and thus low runoff values. All indicators were derived directly from the dynamic simulations with iLand.

**Figure 2:**
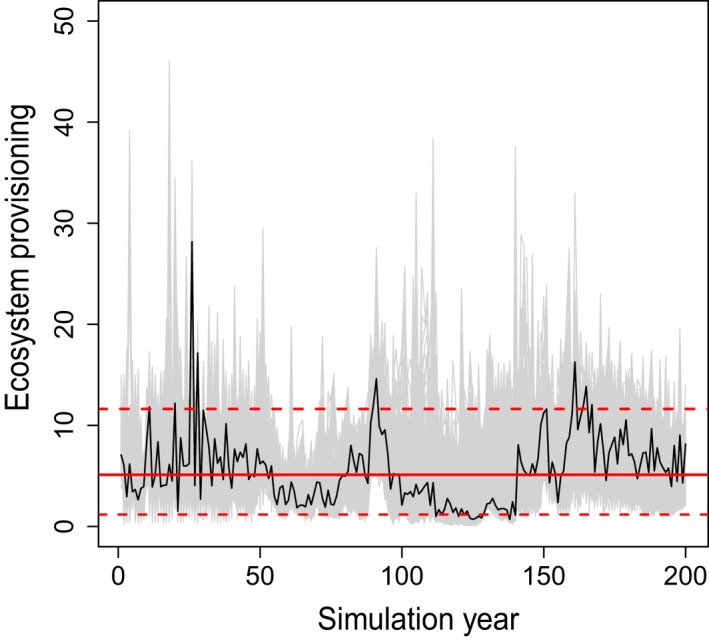
Illustration of deriving temporal stability and level of ecosystem service provisioning from simulated trajectories of ecosystem service indicators (here shown exemplarily for the indicator timber harvest). The black line highlights one simulation run, for which the solid red line indicates the long‐term level of ecosystem service provisioning, here calculated as the time series median. The dashed red lines indicate the 5th and 95th percentile of temporal variation, with the reciprocal value used here to indicate temporal stability of ecosystem service provisioning. The gray lines indicate the envelope of the 479 additional simulated trajectories of the respective indicator, representing different climate scenarios, management strategies, and replications.

To address our first hypothesis of a negative relationship between temporal stability and long‐term level of ES provisioning, these two dimensions were calculated for each indicator and each individual simulation run. Here, it is important to note that both the level of ES provisioning and its temporal stability are emergent properties of our process‐based simulations at the level of individual trees, reflecting the complex interplay between climate (change), soil, management, and natural disturbance. Level of ES provisioning was expressed as the median value over the 200‐yr simulation period. Temporal stability was calculated as one divided by the 5th to 95th percentile range of annual ES estimates over the entire simulation period (Fig. 2, Eq. (1))

(1) $$\text{Temporal stability}=\frac{1}{\left(95\text{th percentile}-5\text{th percentile}\right)}$$

To test for the relationship between ES stability and level of provisioning, a linear regression was calculated across all simulation runs (*n* = 480) individually for each ES indicator. If a significant trade‐off between stability and level of ES provisioning was present, we expected the slope of the regression to be significantly different from zero. The strength of the relationship was assessed using the coefficient of determination (*R* ^2^). To address the question of how diversity influences ES stability, we calculated the exponential Shannon index (Jost 2006) of the tree species in the landscape based on basal area shares. This index represents the effective number of tree species present on the landscape and accounts for both species richness and evenness. Subsequently, the Pearson correlation coefficient was used to analyze the relationship between diversity and ES stability. The R Project for Statistical Computing (R Core Team 2017) was used for the preparation of simulation data and all analyses.

## Results

### Effect of alternative management strategies on the level of ecosystem service provisioning

The level of ecosystem service provisioning varied substantially between management strategies. The spruce‐oriented HIST strategy produced the highest amount of timber. This finding was consistent for both the flow indicator (harvest level) as well as the stock indicator (standing timber volume) used to characterize timber production (Table 2). The BAU strategy maintained similar timber stocks as HIST, but had lower harvest levels. The more broadleaf‐oriented strategies AM1 and AM2 had lower timber stocks and harvest levels over the 200 yr simulation period compared to the spruce‐oriented management strategies BAU and HIST. The broadleaf‐oriented strategies were, however, also considerably less affected by disturbances from wind and bark beetles (Appendix S1: Fig. S18). Consequently, the contribution of salvage harvest to the overall harvest level (Appendix S1: Fig. S19) was considerably lower in AM1 (11.4%) and AM2 (9.1%) compared to HIST and BAU (28.1% and 22.0%, respectively).

**Table 2:** Level of ecosystem service provisioning under alternative forest management strategies

Management strategy	Timber production		Carbon cycling		Site protection	
	Harvest level (m^3^·ha^−1^·yr^−1^)	Timber stock (m^3^/ha)	NEP (Mg C·ha^−1^·yr^−1^)	TEC (Mg C/ha)	Water runoff (mm/yr)	LAI (m^2^/m^2^)
HIST	5.54 (4.76–6.16)	270.5 (244.9–334.8)	0.331 (0.053–0.739)	318.2 (292.3–342.1)	900.2 (883.6–1003.8)	3.58 (3.35–3.89)
BAU	5.34 (4.52–5.99)	260.9 (243.0–321.6)	0.254 (−0.020–0.617)	316.3 (291.6–338.7)	898.7 (884.4–1000.8)	3.50 (3.36–3.85)
AM1	4.80 (4.25–5.47)	225.8 (211.3–267.7)	0.320 (0.030–0.689)	308.9 (282.8–324.7)	902.9 (896.3–1000.8)	3.43 (3.33–3.82)
AM2	4.67 (4.00–5.25)	219.4 (202.8–259.3)	0.327 (0.083–0.774)	311.2 (289.8–332.4)	903.1 (898.8–1001.4)	3.60 (3.50–4.04)

*Notes*

Values are the median over all simulation runs for each strategy with the 5th and 95th percentile range across scenarios and replicates in parentheses. NEP, net ecosystem productivity; TEC, total ecosystem carbon stock; LAI, leaf area index.

The effects of management on carbon cycling were largely congruent with those found for timber production. HIST and BAU had the highest level of total ecosystem carbon (TEC) storage. Under the AM1 strategy, TEC was between 9.3 and 7.4 Mg C/ha lower than under HIST and BAU and 2.3 Mg C/ha lower than under AM2. C uptake (i.e., net ecosystem productivity) was highest under HIST. NEP under BAU management was considerably lower (−0.077 Mg C·ha^−1^·yr^−1^ compared to HIST), while there were no large differences between the other strategies.

The provisioning of site protection against soil erosion was particularly sensitive to the underlying climate scenario. Given that the absolute level of annual precipitation in our study region strongly exceeds the potential evapotranspiration of a fully stocked forest landscape, the amount of water runoff was mainly driven by scenario‐specific differences in precipitation level, with only small effects of management. Runoff was only slightly lower (i.e., indicating better performance regarding site protection) under the spruce‐dominated strategies BAU and HIST compared to the broadleaf‐oriented strategies AM1 and AM2. The second indicator investigated as a proxy for site protection, LAI, showed no functionally relevant differences between strategies (differences of between 0.17 and 0.02 m^2^/m^2^). Considering both indicators, the level of site protection was not affected by management.

### Temporal stability of ecosystem services and its relation to the level of service provisioning

Climate variation and disturbance legacies had a distinct influence on the temporal stability of ES provisioning of the landscape. Climate variation introduced high frequency temporal fluctuation and particularly affected flow variables. Both C uptake and water runoff strongly depended on the climate condition of a given year, and thus showed high interannual variation (Appendix S1: Figs. S21, S22). The legacies of past disturbances, on the other hand, mainly influenced stock variables, and introduced low frequency, multi‐decadal fluctuations into service provisioning trajectories. Here, particularly the uneven forest age distribution and stocking density of our landscape at the beginning of the study period are noteworthy (Appendix S1: Figs. S1, S2). The initial share of large areas of relatively young forests (which are legacies of recent disturbances by wind and bark beetles) resulted in peaks of timber and C stocks as well as LAI in the middle of the 200 yr simulation period, with LAI peaking before timber volume and C stocks (Appendix S1: Figs. S25, S23, S24). Temporal stability of ES provisioning is thus a combined effect of fast (climate variability) and slow (stand development) drivers.

Temporal stability of ES provisioning was distinctly affected by forest management. For timber production, the temporal variation of both indicators was lower in the tree‐species‐rich strategies AM1 and AM2 compared to spruce‐dominated strategies, indicating higher stability of the adaptive management strategies (Table 3). The considerably lower disturbance susceptibility of these strategies (Appendix S1: Fig. S18) is an important factor contributing to their higher temporal stability. Similarly, AM2 had the highest temporal stability in C uptake, and AM1 in C storage. With regard to site protection, BAU and AM1 were temporally most stable. Water runoff performed best under BAU management.

**Table 3:** Temporal stability of ecosystem service provisioning under alternative management strategies

Management strategy	Timber production		Carbon cycling		Site protection	
	Harvest level (m^3^·ha^−1^·yr^−1^)	Timber stock (m^3^/ha)	NEP (Mg C·ha^−1^·yr^−1^)	TEC (Mg C/ha)	Water runoff (mm/yr)	LAI (m^2^/m^2^)
HIST	0.095 (0.076–0.111)	0.00560 (0.00418–0.00668)	0.120 (0.088–0.150)	0.00933 (0.00785–0.01199)	0.00148 (0.00118–0.00150)	0.396 (0.344–0.446)
BAU	0.100 (0.084–0.122)	0.00582 (0.00421–0.00691)	0.126 (0.091–0.157)	0.00872 (0.00734–0.01112)	0.00149 (0.00118–0.00151)	0.449 (0.399–0.487)
AM1	0.111 (0.095–0.132)	0.00724 (0.00483–0.00933)	0.128 (0.093–0.163)	0.01000 (0.00769–0.01305)	0.00145 (0.00117–0.00148)	0.465 (0.390–0.509)
AM2	0.116 (0.099–0.137)	0.00802 (0.00504–0.01055)	0.132 (0.096–0.166)	0.00994 (0.00748–0.01272)	0.00145 (0.00117–0.00147)	0.435 (0.357–0.474)

*Notes*

Values are the median overall simulation runs for each strategy with the 5th and 95th percentile range across scenarios and replicates in parentheses (see Fig. 2 and Eq. (1) for calculation of stability indicator). NEP, net ecosystem productivity; TEC, total ecosystem carbon stock; LAI, leaf area index.

**Figure 3:**
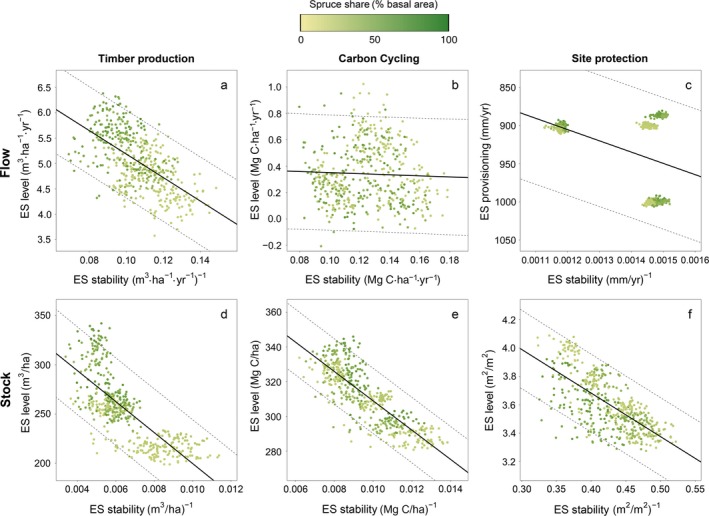
Relationship between temporal stability and level of ecosystem services (ES) provisioning. Flow indicators are shown in the top row and are (a) timber production vs. harvest level, (b) carbon cycling vs. net ecosystem productivity, and (c) site protection vs. water runoff (please note the inverted *y*‐axis), while stock indicators are given in the bottom row and are (d) timber production vs. standing timber volume, (e) carbon cycling vs. total ecosystem carbon stock, and (f) site protection vs. leaf area index. Black lines indicate a regression line, dashed lines are prediction intervals. Colors indicate the mean spruce share of each run, emerging from the dynamic simulations of alternative managements under different climate scenarios. The three distinct clusters shown in panel c are the result of the three climate scenarios studied.

For all ecosystem services, we found a negative relationship between temporal stability and long‐term level of ES provisioning across all studied management strategies and climate scenarios (Fig. 3). Generally, the relationships were more strongly negative for stock indicators than for flow indicators. However, the strength of the trade‐off varied strongly between ecosystem services and individual indicators. The observation period also influenced the strength of the relationship between stability and level of ES provisioning (Appendix S1: Table S5). For timber production, both flow and stock indicators showed a clear negative relationship between the ES stability and ES level (*P* < 0.001, *R* ^2^ = 0.375/0.571, Fig. 3a, d). A 5% increase in harvest level relative to the median harvest level across all scenarios (i.e., an increase by 0.25 m^3^·ha^−1^·yr^−1^) decreased the temporal stability of timber harvest by 10.2%. The effect was even stronger for timber stocks, where a 5% increase relative to the median corresponded to a reduction of 11.8% in stability. Overall, the spruce‐oriented HIST and BAU strategies had a higher level of provisioning but lower stability for both indicators of timber production, compared to the broadleaf‐oriented management strategies.

Temporal stability was also negatively related to long‐term provisioning for indicators of C cycling. Total ecosystem carbon stock (Fig. 3e) showed a strong negative relationship between ES provisioning and stability (*P* < 0.001, *R* ^2^ = 0.674), with a 5% increase in C stocks resulting in a 17.9% reduction in temporal stability. For C uptake, on the other hand, the relationship between temporal stability and ES provisioning was weak and not statistically significant (*P* = 0.367, *R* ^2^ = 0.002).

For site protection, stability and level of ES provisioning were also negatively related, with stronger effects for the stock compared to the flow indicator. Water runoff (Fig. 3c) showed a significant relationship (*P* < 0.001, *R* ^2^ = 0.18), with 5% increase in the median level of ES provisioning associated with 23.0% reduction in ES stability. The influence of the three climate scenarios with their diverging precipitation levels was pronounced, separating the simulation results into three distinct groups. The climate scenario with the highest variability in precipitation (ARPEGE, see Appendix S1: Fig. S6) also had the lowest ES stability. The stock indicator LAI (Fig. 3f) was less sensitive than runoff, with a 5% increase in ES provisioning associated with a 13.5% decrease in ES stability (*P* < 0.001, *R* ^2^ = 0.486). While the relationship between stability and provisioning of ES was stronger for LAI compared to runoff, the relative effect size was higher for runoff.

### Effects of tree species diversity on the temporal stability of ecosystem services

Tree species diversity was positively related to temporal stability for all investigated stock indicators (i.e., standing timber volume, total ecosystem C stock, and LAI). The positive effect of tree species diversity on stability was strongest for timber production, with the stability of both investigated indicators increasing with tree species diversity (Table 3). Also, C stocks were positively related to tree species diversity, while no significant relationship was found for C uptake. For site protection, the diversity–stability relationship showed diverging results for the two indicators investigated: while the temporal stability of LAI increased with tree species diversity, runoff was negatively related to diversity. This is mainly an effect of the different life history traits of the species‐poor (evergreen‐dominated) and species‐rich (deciduous‐dominated) forest types addressed in our study, with the latter having shorter vegetation periods and providing a lower level of site protection during the months of leaf‐off.

## Discussion

### Trade‐offs between stability and level of ecosystem service provisioning

We demonstrated that strong trade‐offs between the stability and level of ecosystem service provisioning exist in temperate forest ecosystems. Our results indicate that increasing the level of service provisioning might at the same time reduce its temporal stability. This generally supports the hypothesis of a negative relationship between the stability and level of ES provisioning. However, the strength of the trade‐off varied with ES and focal indicator. Relationships were generally stronger for stock indicators than for flows, in particular for processes such as C uptake and water runoff. The strong influence of high‐frequency climate variability was weakening a generally negative stability–provisioning relationship for flow indicators. In addition to “fast” (i.e., annual) climate variability, “slow” variability induced by stand development (i.e., at time scales of decades) influenced the temporal stability of ES provisioning. In particular, initial conditions, reflecting the legacies of past disturbances in the form of a skewed age‐class distribution, can have a long‐lasting effect on future ecosystem trajectories (see Appendix S1: Fig. S17 and Temperli et al. 2013) and influence the future stability of ecosystems (Schurman et al. 2018). As a consequence of such slow variability from stand development, we found a change in the trade‐off between stability and level of ecosystem service provisioning over time (Appendix S1: Table S5), underlining that the choice of study period (here 200 yr) can distinctly influence results regarding stability.

Natural disturbances (here wind and bark beetle outbreaks) reduced both the level of ES provisioning as well as its temporal stability. This is in line with a wide range of studies reporting a negative impact of disturbances on ecosystem services (see Thom and Seidl 2016). However, our analysis also revealed considerable complexity in the assessment of disturbance effects on ecosystem service provisioning. While disturbances reduce in situ C storage in the years following an event (Lindroth et al. 2009, Matthews et al. 2017), they can, at the same time, foster C uptake over longer time frames through high growth rates in forests recovering from disturbance (Yue et al. 2016). This underlines the importance of considering both stock and flow indicators and their dynamics over extended time frames in order to comprehensively capture changes in ecosystem services. Similarly, when assuming that disturbed timber is salvaged (as done here), the effect of disturbances on timber production is ambiguous, as they increase the volume of timber harvested, ostensibly increasing the level of ES provisioning. However, such an increase is not necessarily desired in the context of forest management, as salvaging disturbed timber is associated with high harvesting costs, reduced timber quality, and potentially detrimental effects on biodiversity (Prestemon and Holmes 2008, Thorn et al. 2017).

Any findings on ES stability are necessarily contingent on the definition of stability used (Grimm and Wissel 1997). Here, we focused on temporal stability and defined it as the inverse of temporal variability. The advantages of such an aggregate measure of stability are its ease of derivation and considerable information content, as shown in our analysis here. However, whether low stability is the result of short time periods with low ES provisioning, or whether larger‐scale trends in ES trajectories exist remains undetected in such an approach. Alternative approaches could thus consider minimally required thresholds for service provisioning for defining stability, an approach frequently used also when considering ES multifunctionality (Pasari et al. 2013, Ratcliffe et al. 2017). Furthermore, it is important to note that other types of stability besides temporal stability are relevant in the context of managing for ES. Scenario uncertainty (i.e., the variability of ES under different climate scenarios, e.g., Silva Pedro et al. 2015) or the uncertainty with regard to societal preferences for ES (i.e., changes in societally demanded ES, e.g., Seidl and Lexer 2013) are two examples for other types of stability that are also relevant in the context of ecosystem management.

### Stabilizing effects of biodiversity on ecosystem services

We hypothesized a stabilizing effect of high tree diversity on the temporal variation of ecosystem services. This hypothesis is in line with previous findings (Isbell et al. 2011, Harrison et al. 2014, Morin et al. 2014, Silva Pedro et al. 2015, Mori et al. 2017), and follows the “insurance hypothesis”, which states that a higher number of species will stabilize ecosystem functioning as a result of increased response diversity and functional redundancy (Yachi and Loreau 1999). We found general support for this hypothesis, but also showed that such a positive relationship is not universally valid for all ES. The stability of the six ES indicators showed a range of different behaviors in relation to tree species diversity (Table 4). The insurance hypothesis could be generally confirmed for timber production and carbon cycling (see also Silva Pedro et al. 2015). With regard to site protection, we found both positive and negative relationships between diversity and ES stability, depending on the indicator analyzed. This underlines the importance of context when assessing the effects of biodiversity (Ratcliffe et al. 2017, Paquette et al. 2018). It furthermore highlights that a coarse filter approach to increasing ES stability by fostering diversity might not always be successful, requiring more process‐oriented analyses focusing on the ES of local interest.

**Table 4:** Pearson's product‐moment correlation between stability of ecosystem service provisioning and tree species diversity (*n* = 480)

Ecosystem service and type of indicator	Indicator	Correlation coefficient	*P*
Timber production			
Flow	timber harvest level	+0.581	<0.001
Stock	standing timber volume	+0.686	<0.001
Carbon cycling			
Flow	net ecosystem productivity	+0.043	0.346
Stock	total ecosystem C stock	+0.316	<0.001
Site protection			
Flow	water runoff	−0.268	<0.001
Stock	leaf area index	+0.491	<0.001

A factor that complicates the analysis of diversity–stability relationships are the dynamic interactions and feedbacks inherent in forest ecosystems. Natural disturbances, for instance, are a threat to the temporal stability of ecosystem service provisioning, but at the same time foster diversity (Peltzer et al. 2000, Franklin et al. 2002, Silva Pedro et al. 2016), which, in turn, has a generally positive effect on stability. In our study system, for example, bark beetle disturbances are targeting a single host tree species, Norway spruce, and foster the establishment of a variety of other tree species through natural regeneration in the resulting gaps. A simulation run with high disturbance activity will thus have less stable provisioning of ecosystem services, while at the same time tree species diversity is higher than in a scenario with low disturbance activity. This paradox role of disturbances in negatively impacting ecosystem services while simultaneously exerting positive impacts on biodiversity has been reported previously (Thom and Seidl 2016), and needs further attention in the strive toward an improved process‐understanding of the roles of biodiversity in ecosystem service provisioning (Isbell et al. 2017).

A limitation of our analysis in the context of considering the effects of natural disturbances is the focus on two disturbance agents, wind and *Ips typographus*. Notwithstanding the fact that these two agents are currently the by far most important disturbance agents in Central Europe (Schelhaas et al. 2003, Thom et al. 2013), other agents could increase in importance as climate and tree species composition change in the future. Of particular concern in this context are invasive alien tree pests, which have the potential to strongly alter forest ecosystems and the services they provide (Liebhold et al. 2017, Seidl et al. 2018b). Future studies focusing on the temporal stability of ES provisioning in forests should thus not only focus on the effects of climate change and natural disturbance agents, but also consider the effects of invasive alien pests explicitly (Pautasso et al. 2010).

### Implications for forest management

In the face of an increasingly uncertain future, forest management needs approaches that robustly provide ecosystem services under a wide range of possible climate and disturbance regimes (Millar et al. 2007, Daniel et al. 2017). Our results show that increasing stability may come at the expense of a reduced level of ES provisioning. In other words, achieving a temporally stable *and* maximum ES supply will often not be simultaneously possible in ecosystem management. It is thus important that studies optimizing ES supply (Diaz‐Balteiro et al. 2017, Mina et al. 2017, Triviño et al. 2017) increasingly account not only for the level of ES provisioning but also for its temporal stability (for example by introducing minimum levels of ES provisioning required, cf. Härtl et al. 2016). In the context of our study landscape, we found that management strategies fostering a diverse, future‐adapted portfolio of tree species in combination with silvicultural measures reducing risks (such as shortening rotations periods and increasing thinning frequency) resulted in a more stable provisioning of the majority of the ecosystem services considered (see also Temperli et al. 2012). However, considering both the stability and level of ES provisioning, we did not find a clear best practice management strategy, which could provide both a high level of service provisioning and a temporally stable trajectory of ES. In the context of operational decision making managers will thus have to identify a locally appropriate mix between level and stability of ES, based on the social‐ecological context and their personal level of risk aversion (Blennow et al. 2014).

Our results indicate that a higher level of ES supply can be achieved by management strategies taking higher risks. Yet it is important to note that we did not explicitly consider the fact that stability is of varying importance in the management of different ES, and that hierarchical dependencies may exist. In the context of the services considered here, for instance, stability is more important for site protection than for carbon cycling or timber production. Already, a relatively short exposure to open canopy conditions can lead to a significant loss of soil (Morris and Moses 1987, Reger et al. 2015), which in turn would have strong negative effects on timber production and carbon cycling through reduced site productivity. In many cases, stability might thus be more important than the level of ES provisioning for maintaining the capacity of ecosystems over long time frames. Our finding of significant trade‐offs between stability and level of ES provisioning thus suggests that previous studies focusing solely on the latter might overestimate the ES supply that is realistically achievable from ecosystems. We thus suggest that considerations of stability should be included more explicitly in future assessments of ecosystem service supply.

## Data Availability

Data are available on Figshare: https://doi.org/10.6084/m9.figshare.6820172

## Note

1
http://iland.boku.ac.at/
