# Supplementary material for: Trade‐offs between temporal stability and level of forest ecosystem services provisioning under climate change
Source: Ecol Appl. 2018 Sep 4;28(7):1884–96. doi: 10.1002/eap.1785 (PMC6378588; doi:10.1002/eap.1785)
Supplement: Supplementary file 1 [file EAP-28-1884-s001.pdf]

Katharina Albrich, Werner Rammer, Dominik Thom, Rupert Seidl:
Trade-offs between temporal stability and level of forest ecosystem services provisioning under climate change
Ecological Applications

APPENDIX S1

Section S1. Initial Conditions and Drivers

S1.1. Current Vegetation

To initialize the vegetation in the simulations, we made use of several data sources. Management plans for the period 2010-2020 by the current stewards of the landscape (Austrian Federal Forests) provided stand-level estimates of stocking volume (Figure S1) per tree species and age class (Figure S2). In total, 1686 stands with an average stand area of 3.4 ha were initialized. Data from a plot-level inventory (262 sample points inventoried in 2004 and 2005) provided additional information on the characteristics of individual trees (height, diameter at breast height). Individual trees were then iteratively sampled to meet the stand-level characteristics described in the management plans.

As a third data source, Airborne Laser Scanning Data recorded between 2009 and 2013 (Figure S3), with a horizontal resolution of 1 m (southern part of the landscape) and 0.5 m (northern part) provided information on vertical (e.g. tree heights) and horizontal (position of individual trees, canopy gaps) stand structure. Additionally, aerial photographs were used to identify areas not suitable for tree growth (e.g. rocky outcrops), which were then excluded from the simulated forest area.

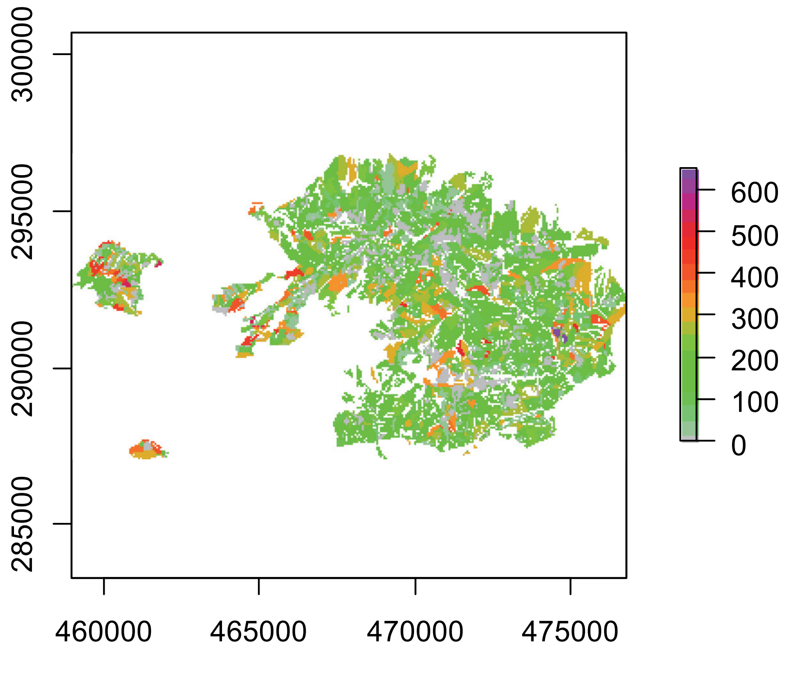

Figure S1: Volume in m^3 ha^{-1} per stand according to management plans

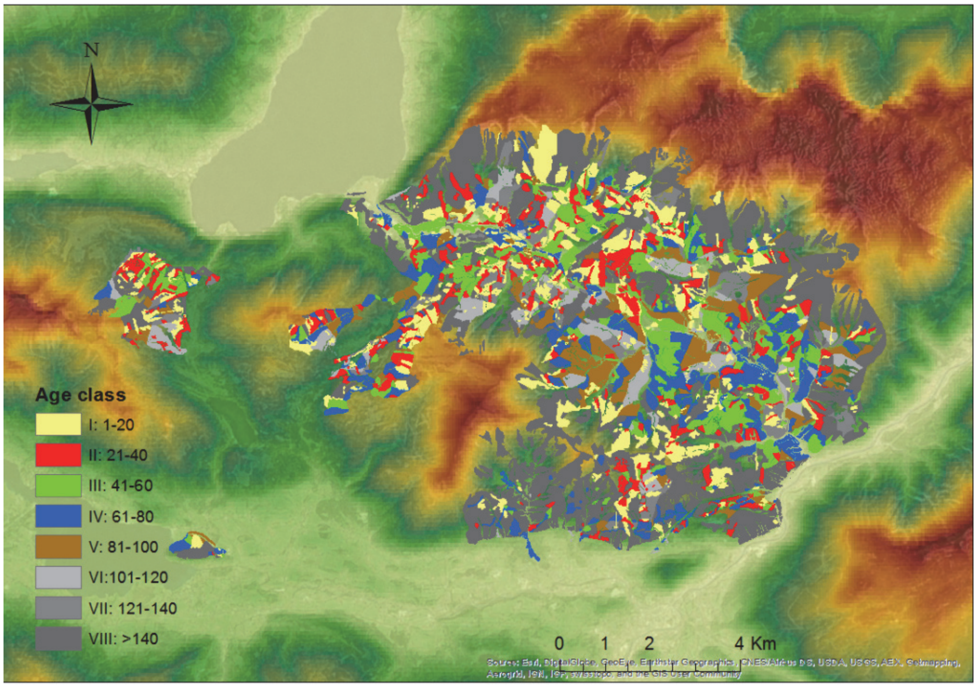

Figure S2: Age classes according to management plans

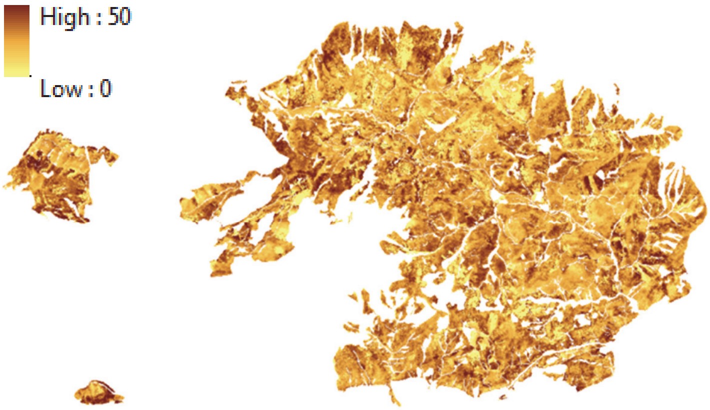

Figure S3: Lidar canopy height on a 10x10m grid in m.

S1.2. Soil

Soil data was prepared on a 100x100 m grid. A site classification by the Austrian Federal Forests (Weinfurter 2004) provided spatially explicit information on relative site quality. To derive quantitative information regarding soil type, soil depth, soil carbon and plant available nitrogen, we

made use of data from the Austrian Forest Soil Survey AFSS (Seidl et al. 2009). Based on stratification by site type and elevation, the grid cells in the simulation area were imputed with information from matching AFSS sample plots. To assess soil texture, we used soil inventory data from the Kalkalpen National Park (see also Thom et al. 2017a) which is geographically close and has similar geologic and pedologic features for soil types occurring in both areas. For soil types not present at the National Park, data from Leitgeb et al. (2013) were used to derive soil texture. Soil depth was reduced to effective soil depth by subtracting rock fraction, derived from Leitgeb et al. (2013). The mean effective soil depth over the entire landscape was 200 ± 74 mm, with a sand content of on average 31.2 ± 18.9% and annual plant-available nitrogen of 49.5 ± 3.5 kg ha^{-1} yr^{-1}.

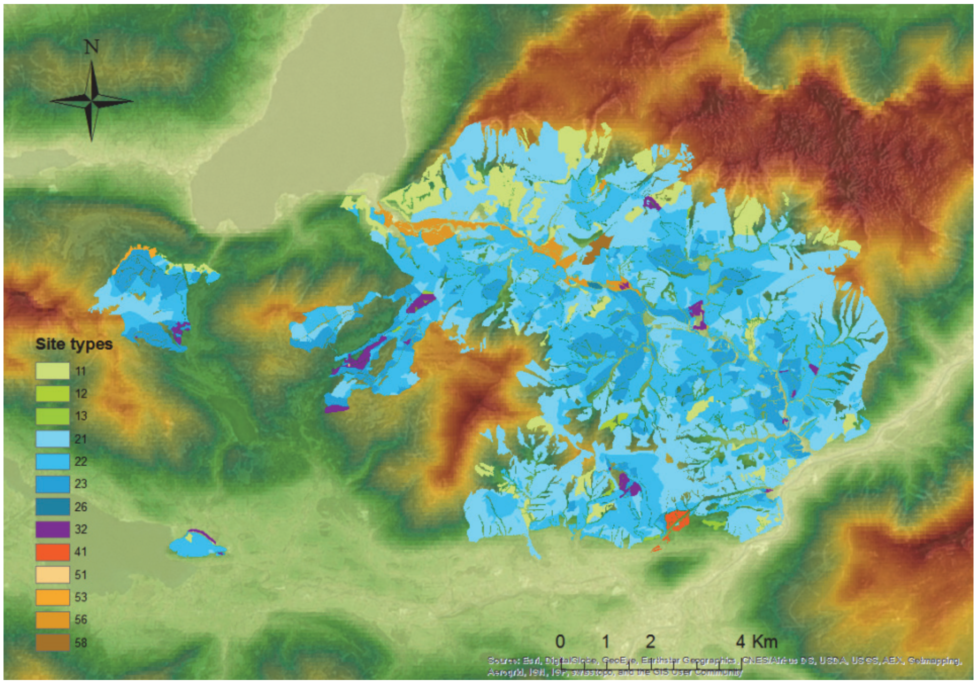

Figure S4: Site types according to the definition of Weinfurter (2004). Higher numbers indicate more productive sites

<u>S1.3 Climate</u>

Three climate change scenarios were derived from different combinations of global and regional circulations models under the A1B emission scenarios (Nakicenovic et al. 2000). Previous analyses (Thom et al. 2017b) have shown that the projected changes in temperature and precipitation for those scenarios lie within the range of the newer RCP4.5 and RCP6.0 scenarios. The GCM-RCM combinations used were CNRM-RM4.5 driven by ARPEGE (Radu et al. 2008), referred to as ARPEGE, MPI-REMO (Jacob 2001), abbreviated as REMO and ICTP-RegCM3, ICTP for short, both driven by ECHAM5 (Pal et al. 2007).

The climate change scenarios assume an increase in the mean annual temperature of 3.2 – 3.3°C (Fig. S5) and a change in mean annual precipitation of -84 to +160 mm until the end of the 21st century

relative to the baseline period 1950-2010 (Fig. S6). The climate data was downscaled statistically to a 100x100 m resolution, and daily time series of minimum and maximum temperature, precipitation, radiation and vapor pressure deficit (vpd) were produced as input data for the model.

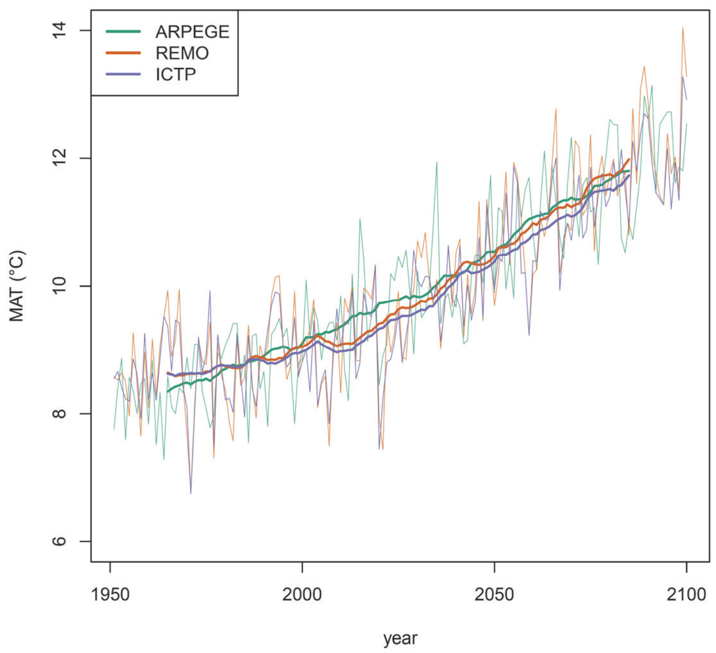

Figure S5: Mean annual temperature in the three different climate scenarios for an exemplary 100 x 100m cell of the landscape. ARPEGE=CNRM-RM4.5 driven by ARPEGE, REMO=MPI-REMO, driven by ECHAM5, ICTP=ICTP-RegCM3, driven by ECHAM5)

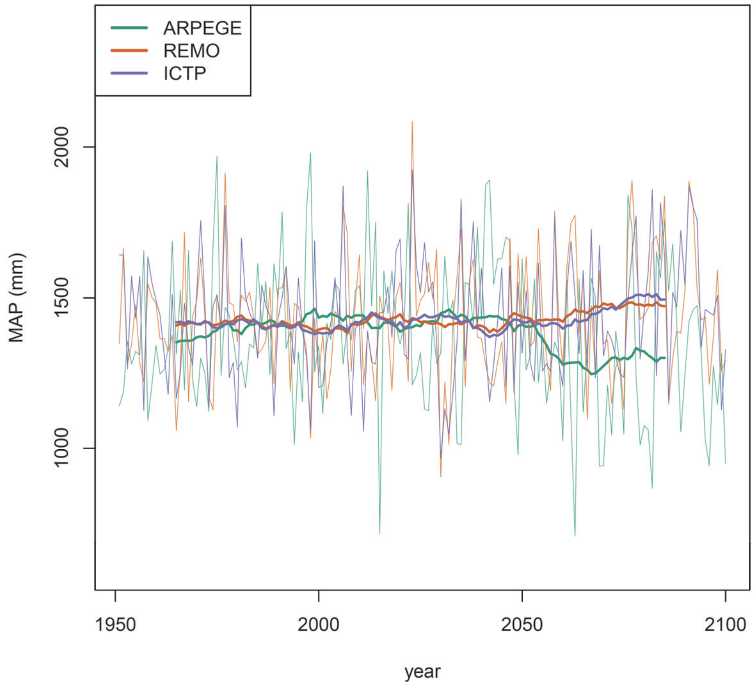

Figure S6: Mean annual precipitation under the three different climate scenarios for an exemplary 100 x 100m cell of the landscape. ARPEGE=CNRM-RM4.5 driven by ARPEGE, REMO=MPI-REMO, driven by ECHAM5, ICTP=ICTP-RegCM3, driven by ECHAM5)

S1.4. Management

To adapt the four management strategies to the stand level, accounting for differences in climate and site conditions throughout the landscape, we translated them into eight different stand treatment programs (stp). Stps were assigned to stands according to site quality, aspect and elevation (see Table S1 for the assignment variables and Figure S7 for the spatial distribution of stps). For each stp, target species composition (at the end of the rotation period) and planted species composition (Table S2) were defined, and silvicultural treatments (tendings and thinnings) as well as the rotation period were specified (Table S3). Final harvests were executed as clearcuts.

Table S1: Assignment of stand treatment programs. Sites were separated according to site type (L=low productivity, H=high productivity), aspect, and slope location ("lower" slopes represent nutrient and water accumulating sites, "upper" slopes signify an export of nutrient and water)

Stand Treatment Programme	Site type	Aspect	Slope position	Area [ha]	% of landscape
stp01	L	N	upper	391.36	6.79
stp02	L	N	lower	1537.31	26.68
stp03	L	S	upper	838.87	14.56
stp04	L	S	lower	2027.56	35.97
stp05	H	N	upper	2.44	0.04
stp06	H	N	lower	553.12	9.60
stp07	H	S	upper	10.04	0.17
stp08	H	S	lower	355.44	6.17

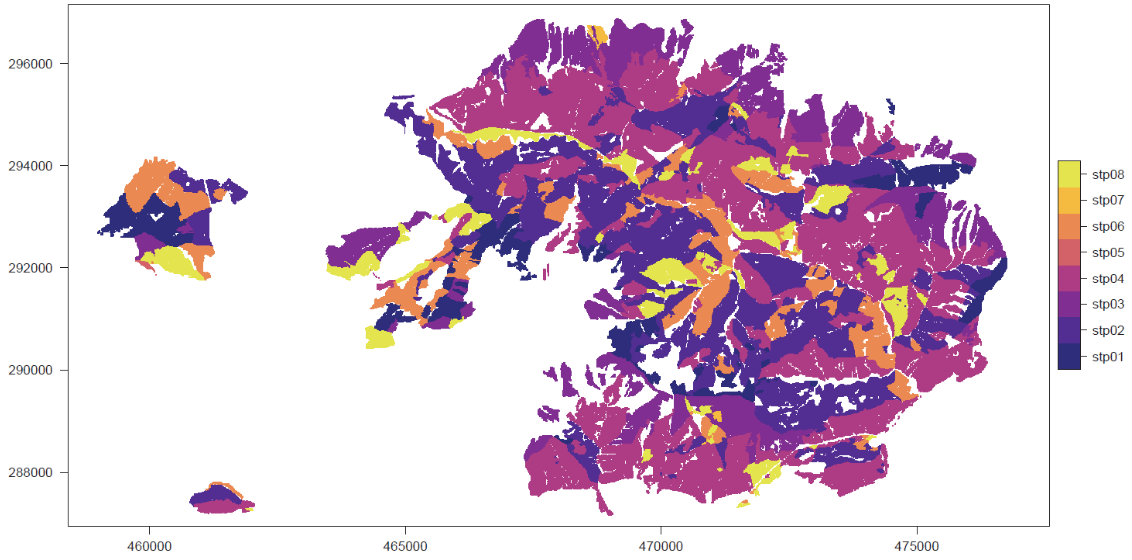

Figure S7: Spatial distribution of stand treatment programs (stp).

Table S2: Target tree species composition (at the end of a rotation period) and planted species shares for each stand treatment program. BAU, HIST, AM1 and AM2 represent the four management alternatives. piab: Picea abies, lade: Larix decidua, fasy: Fagus sylvatica, abal: Abies alba, pisy: Pinus sylvatica, qupe: Quercus petrea, qusp: Quercus sp.

**STP**	**Target species composition**				**Planted species**			
	BAU	HIST	AM1	AM2	BAU	HIST	AM1	AM2
stp01	0.3 piab, 0.3 lade, 0.4 fasy	1.0 piab	0.3 piab, 0.4 lade, 0.2 fasy, 0.1 abal	0.1 piab, 0.7 fasy, 0.1 abal, 0.1 pisy+qusp	0.3 piab, 0.3 lade, 0.4 fasy	1.0 piab	0.4 lade, 0.2 fasy, 0.1 abal	0.7 fasy, 0.1 abal, 0.05 pisy, 0.05 qupe
stp02	0.4 piab, 0.3 lade, 0.3 fasy	1.0 piab	0.3 piab, 0.4 lade, 0.2 fasy, 0.1 abal	0.1 piab, 0.6 fasy, 0.1 abal, 0.1 pisy, 0.1 qusp	0.3 piab, 0.3 lade, 0.4 fasy	1.0 piab	0.4 lade, 0.2 fasy, 0.1 abal,	0.6 fasy, 0.1 abal, 0.05 pisy, 0.05 qupe
stp03	0.3 piab, 0.2 lade, 0.5 fasy	1.0 piab	0.1 lade, 0.6 fasy, 0.1 abal, 0.1 pisy	0.2 piab, 0.6 fasy, 0.2 abal, 0.1 abal, 0.1 pisy+qusp	0.3 piab, 0.3 lade, 0.4 fasy	1.0 piab	0.1 lade, 0.6 fasy, 0.1 abal, 0.2 pisy	0.6 fasy, 0.1 abal, 0.05 pisy, 0.05 qupe (patch
stp04	0.7 piab, 0.1 lade 0.2 fasy	1.0 piab	0.1 lade, 0.6 fasy, 0.1 abal, 0.1 pisy	0.1 piab, 0.5 fasy, 0.1 abal, 0.1 pisy, 0.2 qusp	0.3 piab, 0.3 lade, 0.4 fasy	1.0 piab	0.1 lade, 0.6 fasy, 0.1 abal, 0.2 pisy	0.5 fasy, 0.1 abal, 0.1 pisy, 0.2 qupe
stp05	0.7 piab, 0.1 lade 0.2 fasy	1.0 piab	0.5 piab, 0.2 lade, 0.2 fasy, 0.1 abal	0.1 piab, 0.7 fasy, 0.1 abal, 0.1 pisy+qusp	0.6 piab, 0.2 lade, 0.2 fasy	1.0 piab	0.2 lade, 0.1 fasy, 0.1 abal	0.7 fasy, 0.1 abal, 0.05 pisy, 0.05 qupe
stp06	0.7 piab, 0.1 lade 0.2 fasy	1.0 piab	0.5 piab, 0.2 lade, 0.2 fasy, 0.1 abal	0.1 piab, 0.6 fasy, 0.1 abal, 0.1 pisy, 0.1 qusp	0.6 piab, 0.2 lade, 0.2 fasy	1.0 piab	0.2 lade, 0.1 fasy, 0.1 abal	0.6 fasy, 0.1 abal, 0.05 pisy, 0.05 qupe
stp07	0.7 piab, 0.1 lade 0.2 fasy	1.0 piab	0.3 piab, 0.1 lade, 0.5 fasy, 0.1 abal	0.1 piab, 0.6 fasy, 0.1 abal, 0.1 pisy, 0.1 qusp	0.6 piab, 0.2 lade, 0.2 fasy	1.0 piab	0.1 lade, 0.5 fasy, 0.1 abal	0.6 fasy, 0.1 abal, 0.05 pisy, 0.05 qupe
stp08	0.7 piab, 0.1 lade 0.2 fasy	1.0 piab	0.3 piab, 0.1 lade, 0.5 fasy, 0.1 abal	0.1 piab, 0.6 fasy, 0.1 abal, 0.1 pisy, 0.1 qusp	0.6 piab, 0.2 lade, 0.2 fasy	1.0 piab	0.1 lade, 0.5 fasy, 0.1 abal	0.6 fasy, 0.1 abal, 0.05 pisy, 0.05 qupe

Table S3: Approximate scheduling for prescribed silvicultural activities (thinnings, tendings, final harvest) within stand treatment programs. Actual scheduling is done dynamically within the model. Treatment times are given as years since stand establishment.

STP	Rotation period (years)				Number of thinnings				Thinning age (years)				Number of tendings				Tending age (years)			
	BAU	HIST	AM1	AM2	BAU	HIST	AM1	AM2	BAU	HIST	AM1	AM2	BAU	HIST	AM1	AM2	BAU	HIST	AM1	AM2
stp01	140	140	120	120	1	1	2	2	60	60	30/60	30/60	1	1	1	1	10	10	10	10
stp02	130	130	120	120	2	2	2	2	40/60	40/60	30/60	30/60	1	1	1	1	10	10	10	10
stp03	140	140	120	120	1	1	2	2	60	60	30/60	30/60	1	1	1	1	10	10	10	10
stp04	130	130	120	120	2	2	2	2	40/60	40/60	30/60	30/60	1	1	1	1	10	10	10	10
stp05	120	120	120	120	2	2	2	2	40/60	40/60	30/60	30/60	2	2	1	1	2/10	2/10	10	10
stp06	120	120	120	120	2	2	2	2	40/60	40/60	30/60	30/60	2	2	1	1	2/10	2/10	10	10
stp07	120	120	120	120	2	2	2	2	40/60	40/60	30/60	30/60	2	2	1	1	2/10	2/10	10	10
stp08	120	120	120	120	2	2	2	2	40/60	40/60	30/60	30/60	2	2	1	1	2/10	2/10	10	10

Section S2. Evaluation and Testing

S2.1 Forest Growth and Composition

S2.1.1. Productivity

Productivity was tested against independent growth data from yield tables, determined based on field observations as recorded in the forest management plan. For a representative subset (n=490) of simulated 100 x 100m cells, model results were compared to yield class information from the management plans. Tests were done mimicking the yield tables underlying the management plan data with the model, simulating pure stands of 1 ha for 100 years from bare ground, with management prescribed as in the respective yield tables. Figure S8 shows a landscape overview of mean annual increment (a), dominant height (b), and mean diameter (c) for the most common tree species. Productivity testing was done under past (1951-2010) climate.

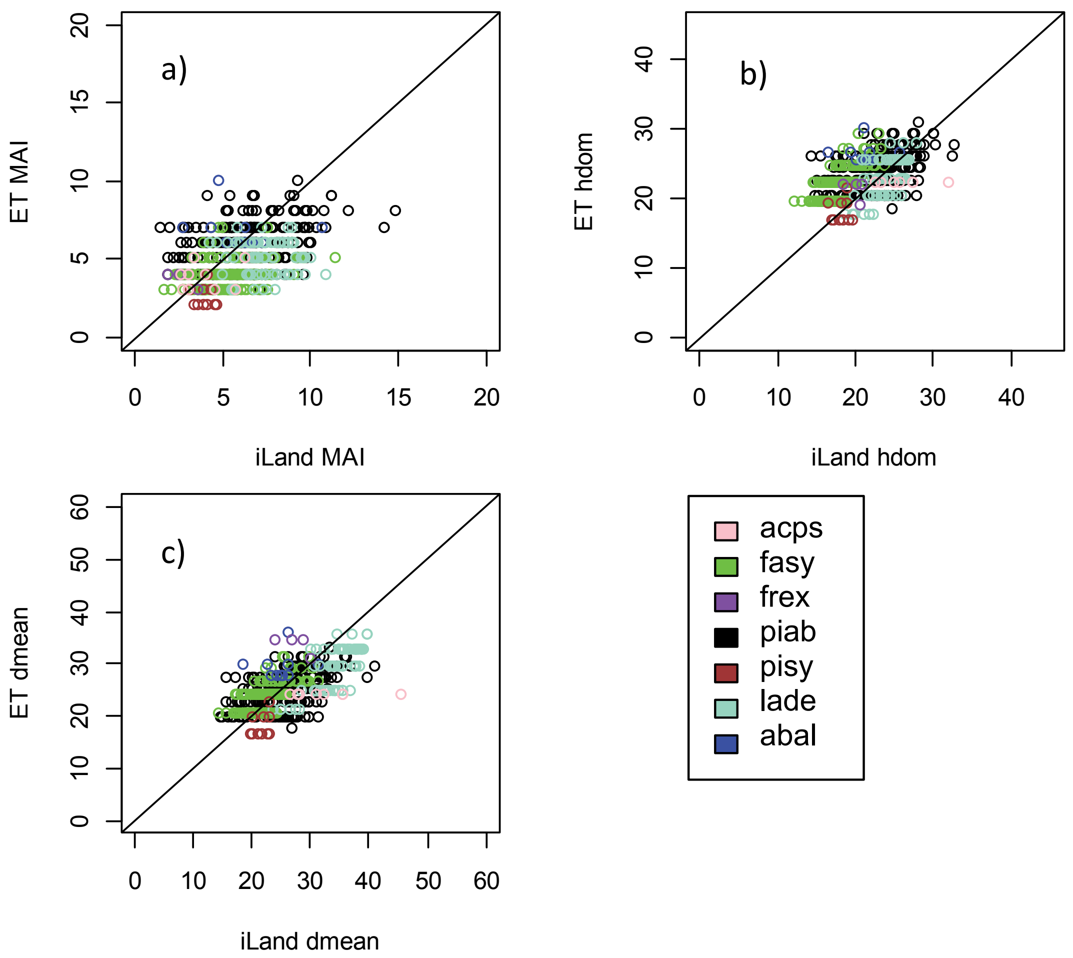

Figure S8: Evaluation of Productivity. The x-axis (iLand simulations) represents the simulation results, the y-axis (ET) shows data from yield tables. Presented are the mean annual increment (a), the dominant height (b) and the mean diameter (c) of a pure 1 ha stand at the age of 100 years. acps=acer pseudoplatanus, fasy=fagus sylvatica, frex=fraxinus excelsior, piab=picea abies, pisy=pinus sylvestris,

S2.1.2 Potential natural vegetation

Simulations from bare ground were run for 2500 years under static past (1951-2010) climate, and the resulting vegetation composition (Figure S9, Figure S10) was compared to descriptive accounts of the potential natural vegetation composition (Kilian et al. 1994). For the northern part of the landscape, a detailed map and description of the potential natural vegetation was available from field surveys (Frank, 1992, Figure S11). The simulation results matched expectations satisfactorily, with beech-forest types dominating low- to mid-elevation areas and mixed spruce-fir-beech forests in higher elevation areas (cf. Kilian et al. 1994).

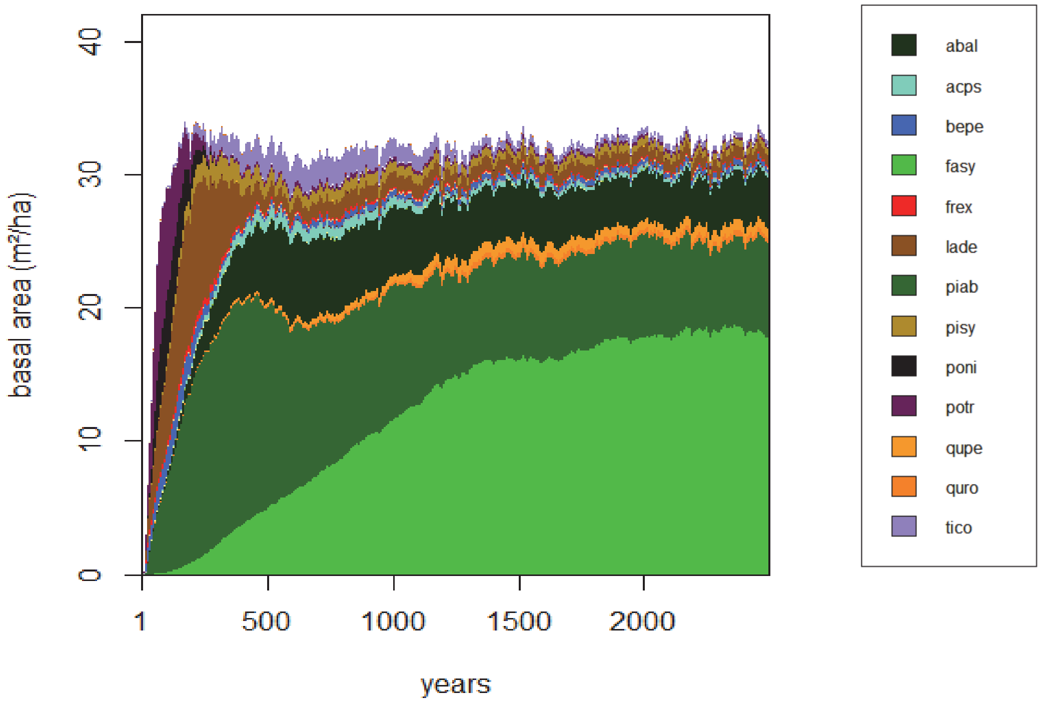

Figure S9: Species composition on the landscape over 2500 years of simulation from bare ground under static climate. abal=abies alba, acps=acer pseudoplatanus, bepe=betula pendula, fasy=fagus sylvatica, frex=fraxinus excelsior, lade=larix decidua, piab=picea abies pisy=pinus sylvestris, poni=populus nigra, potr=populus tremula, qupe=quercus petrea, quro=quercus robur, tico=tilia cordata)

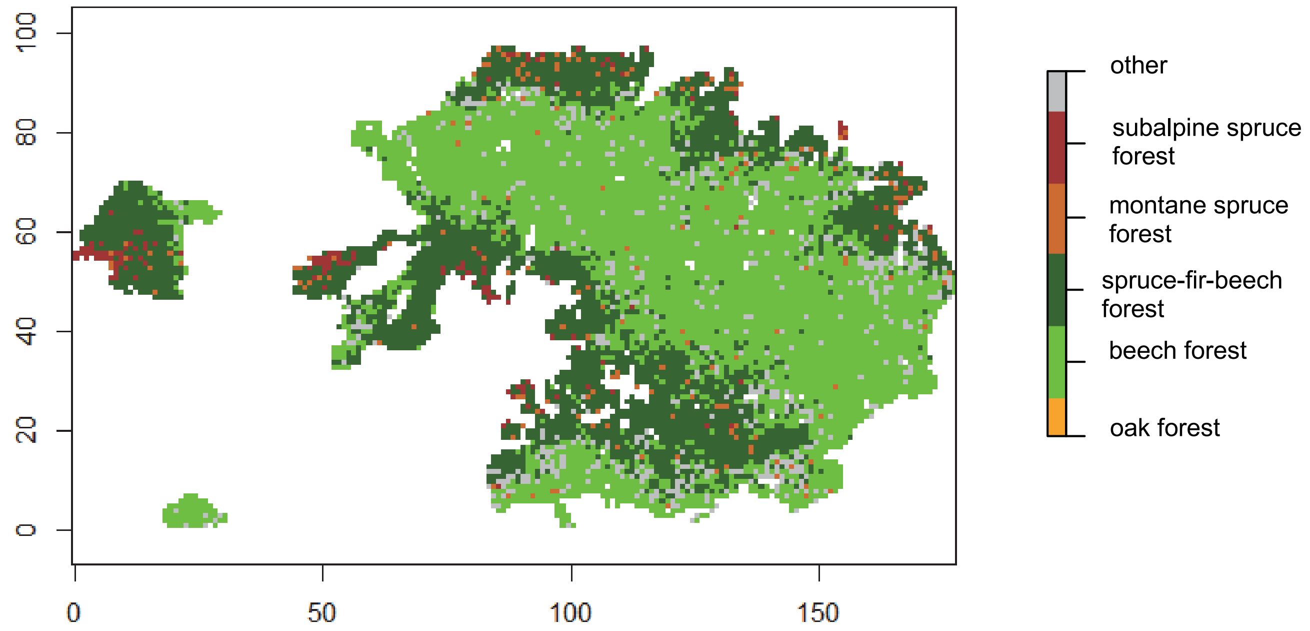

Figure S10: Spatial distribution of forest types in the potential natural vegetation after 2500 years of simulation. Each cell indicates the simulated forest type for 1ha. Forest types were classified according to Lexer et al. (2002).

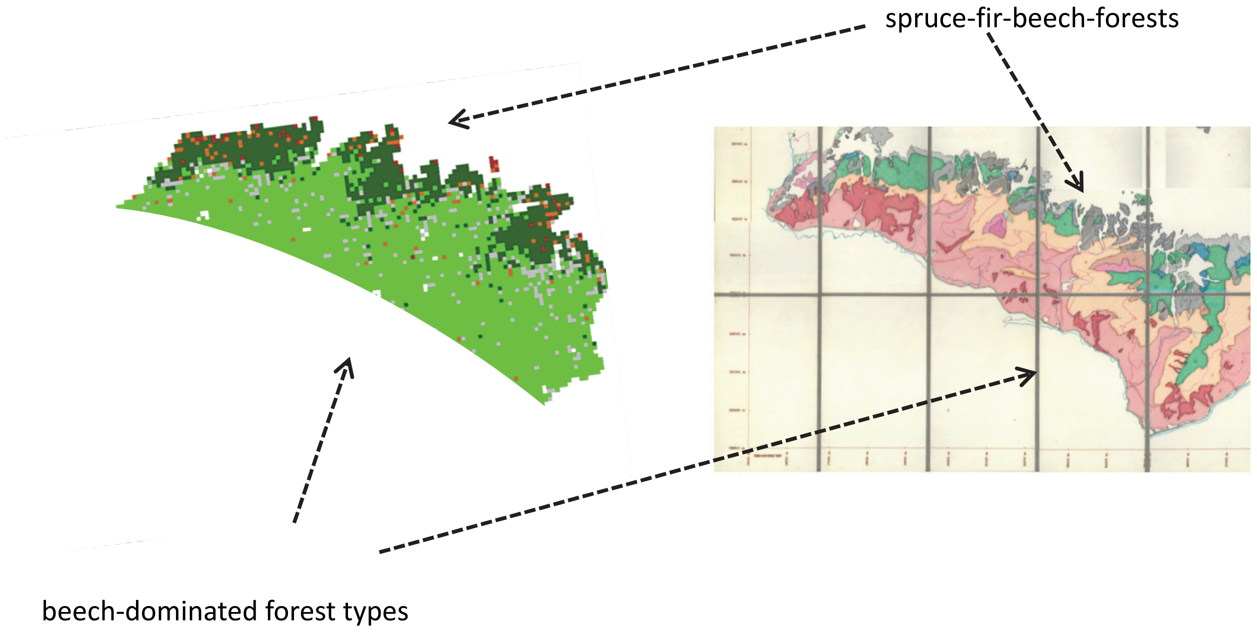

Figure S11: Comparison between simulated potential vegetation and a field-based mapping by Frank (1992) for the north-eastern part of the Weissenbachtal.

S2.1.3. Management:

The implementation of the management strategies was tested on the level of individual stands and stand treatment programs as well as on the landscape scale. We tested the proper implementation of management, focusing on the sustainable harvest constraint, i.e. planned and realized harvests must not exceed the annual increment (Figure S12). We also analyzed the ability of ABE to steer stand development toward the prescribed management goals. Figure S13 presents a comparison between realized and prescribed target tree species shares. The evaluations were done over 200 years under static past climate.

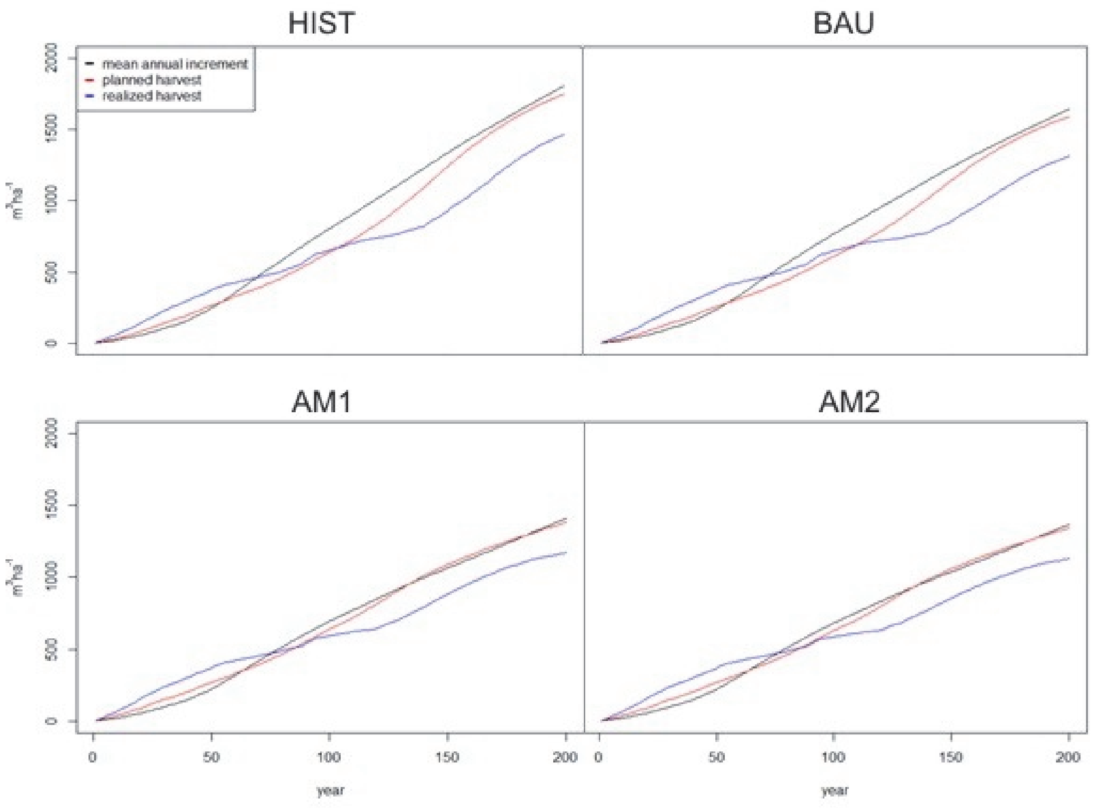

Figure S12: Test of the sustainable harvest constraint in the simulated management strategies. The realized (blue) and planned harvest levels (red) in ABE are compared to the mean annual increment (black). All indicators are shown as cumulative sum over the simulation period. Initial overshooting (realized harvests exceeding mean annual increments) is due to the presence of many old stands at the beginning of the simulation.

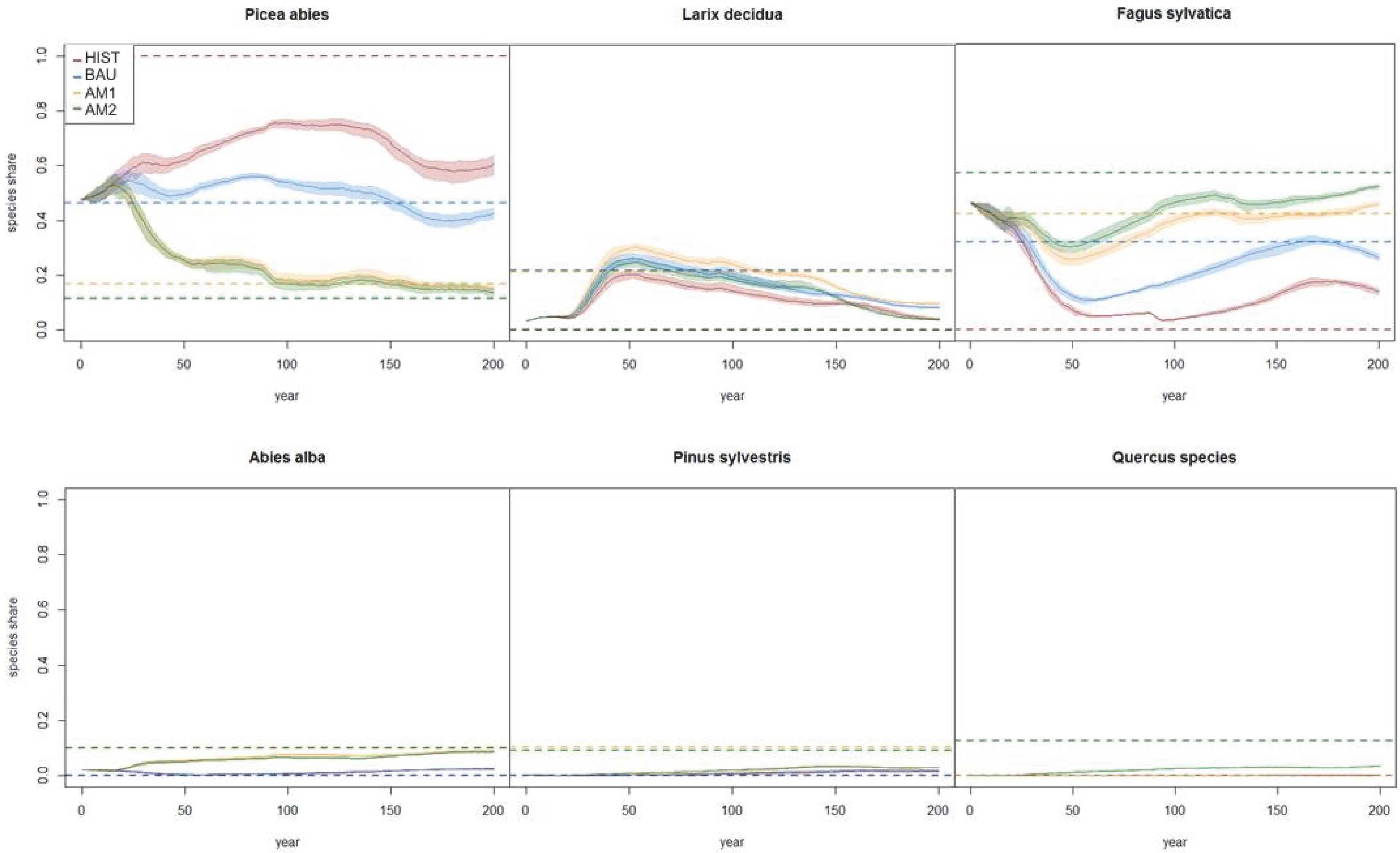

Figure S13: Development of tree species shares under different management strategies. Dashed lines specify tree species targets; continuous lines represent mean simulated species shares, the enveloping bands represent the 5th -95th percentiles.

S2.2. Carbon Cycle

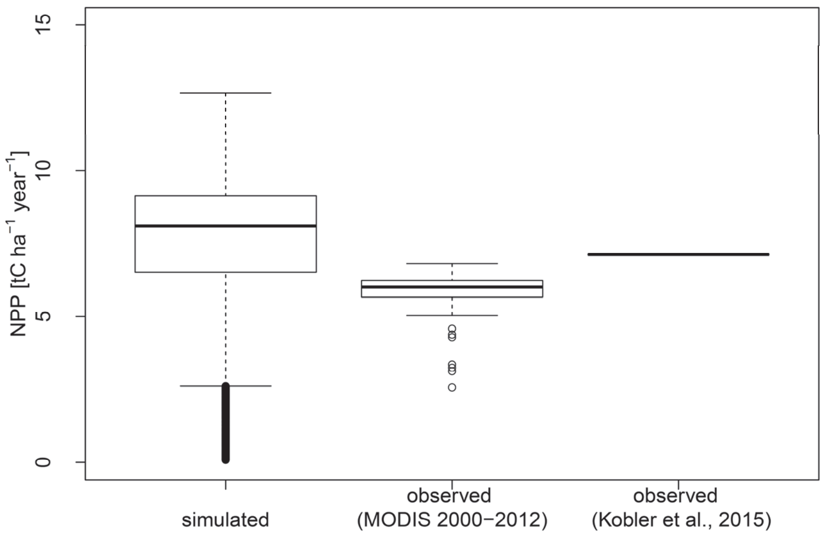

Figure S14: Comparison of simulated NPP (average over the 200 year simulation period for 1 ha pixels under baseline climate, historical disturbances and BAU management) to NPP derived from MODIS (1 km horizontal resolution, period 2000-2012) for the study area (Neumann et al. 2016) as well as to a stand-scale observation on the nearby LTER site Zöbelboden (Kobler et al. 2015).

S2.3. Water Cycle

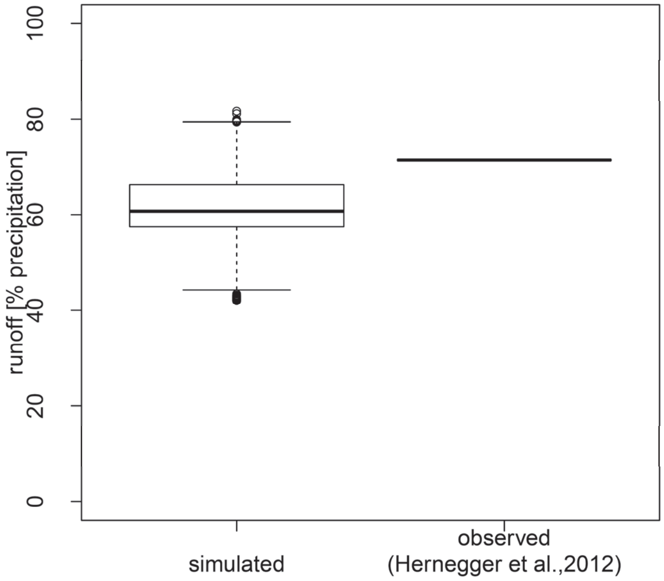

Figure S15: Comparison between simulated runoff in % of precipitation (annual values for the landscape over the 200 year simulation period for 1 ha pixels under baseline climate, historical disturbances and BAU management) and observed mean values for a hydrologically similar catchment (upper Enns river, Austria). Note that the reference catchment is only 55 % forested (Herrnegger et al. 2012), while simulated data are for the forest portion of Weissenbachtal.

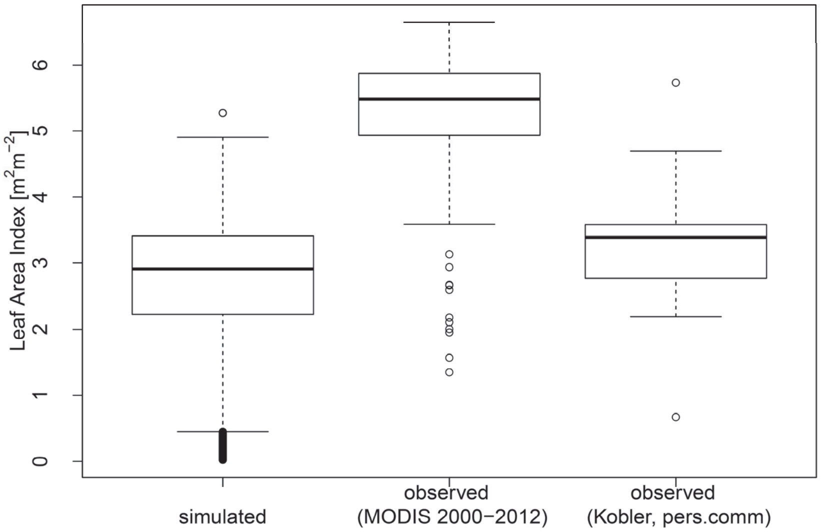

Figure S16: Comparison of simulated Leaf Area Index (average over the simulation period for 1 ha pixels under baseline climate, historical disturbances and BAU management) to LAI derived from MODIS observations (period 2000-2012) for the study area (Yang et al. 2004) and a set of exploratory LAI measurements from nearby LTER site Zöbelboden (Johannes Kobler, personal communication, April 5, 2018) . Note that simulated data refer to the LAI of trees >4m height only, while MODIS NPP is the LAI of the complete vegetation (including forest floor vegetation, which is an important component in these forest types, see Zehetgruber et al. (2017). Measurements from the LTER site are of the tree canopy.

Section S3. Supplementary results

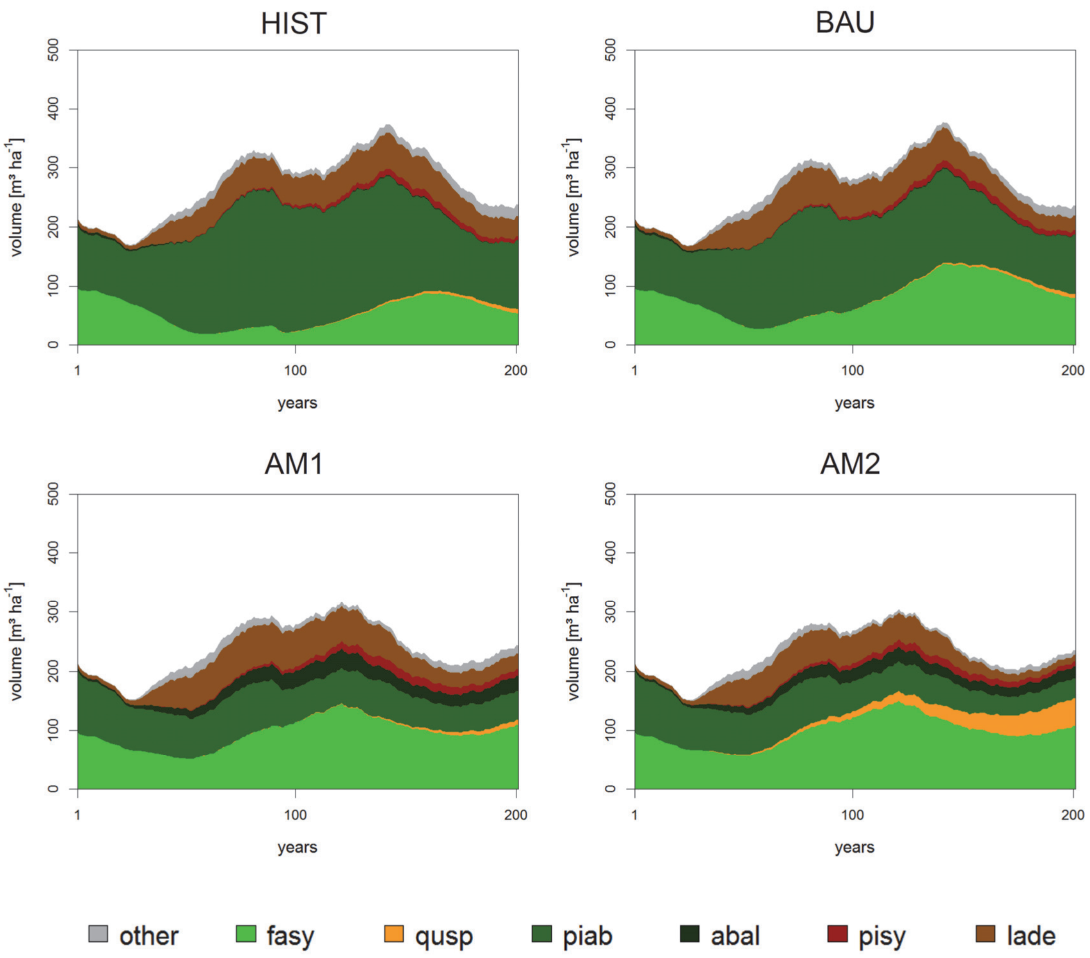

Figure S17: Volume and species composition over time under the four management strategies. The volume is aggregated over all climate scenarios and disturbance scenarios and replicates.

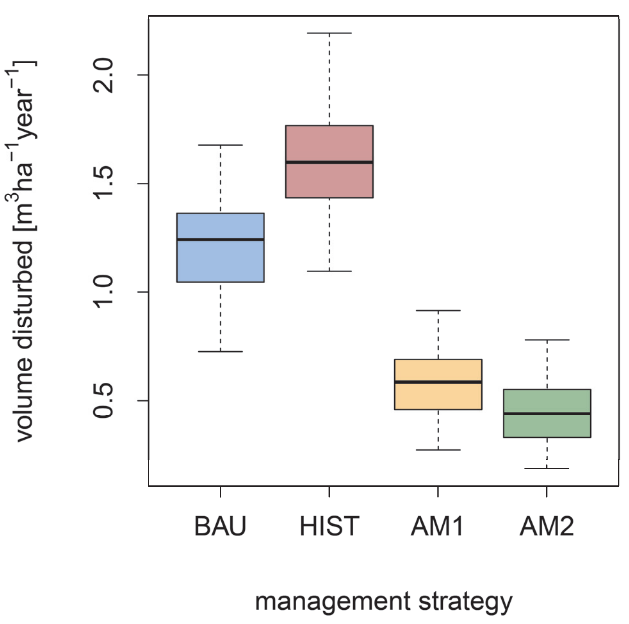

Figure S18: Average timber volume disturbed per management strategy (median over all simulation years).

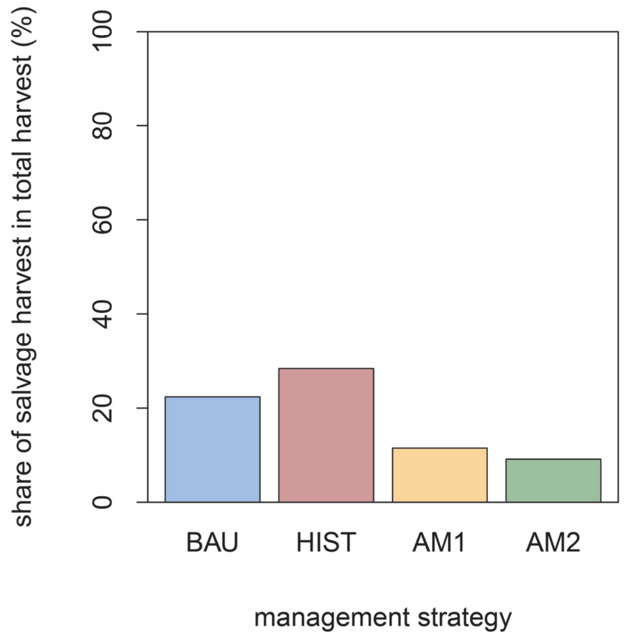

Figure S19: Share of salvage harvest in percent of total harvest (median over all climate scenarios and simulation years).

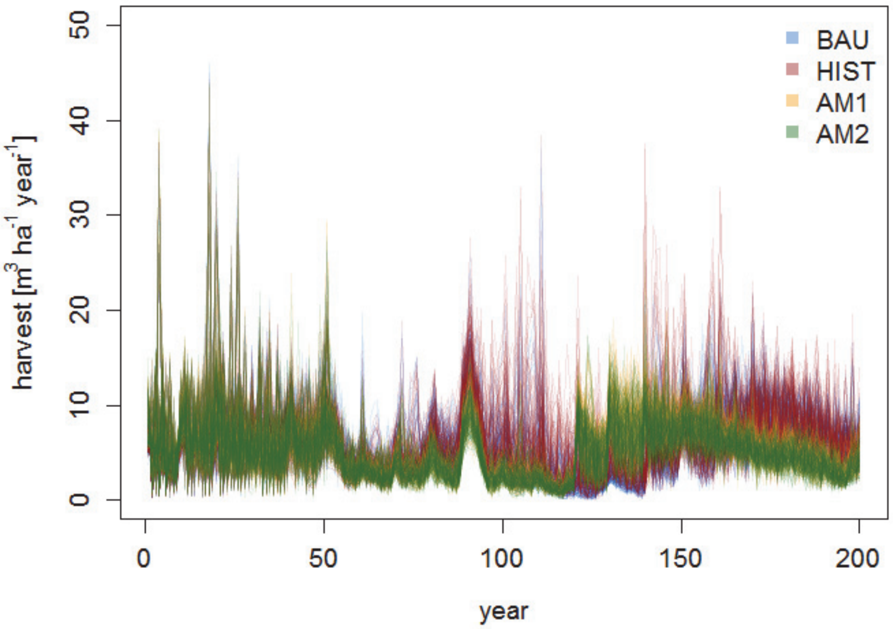

Figure S20: ES indicator timber flow (harvest) over the entire simulation period. Each line represents one simulation run.

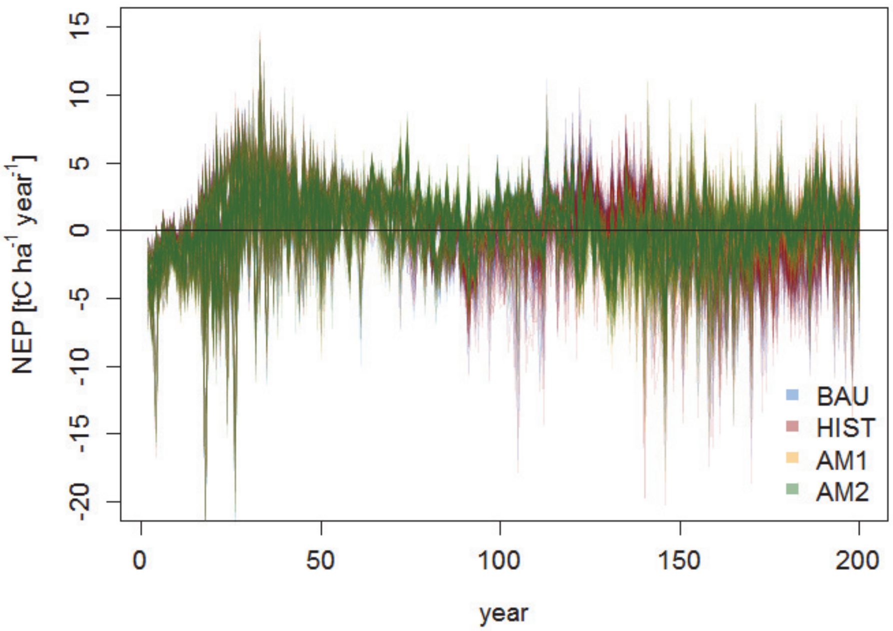

Figure S21: ES indicator carbon flow (NEP) over the entire simulation period. Each line represents one simulation run.

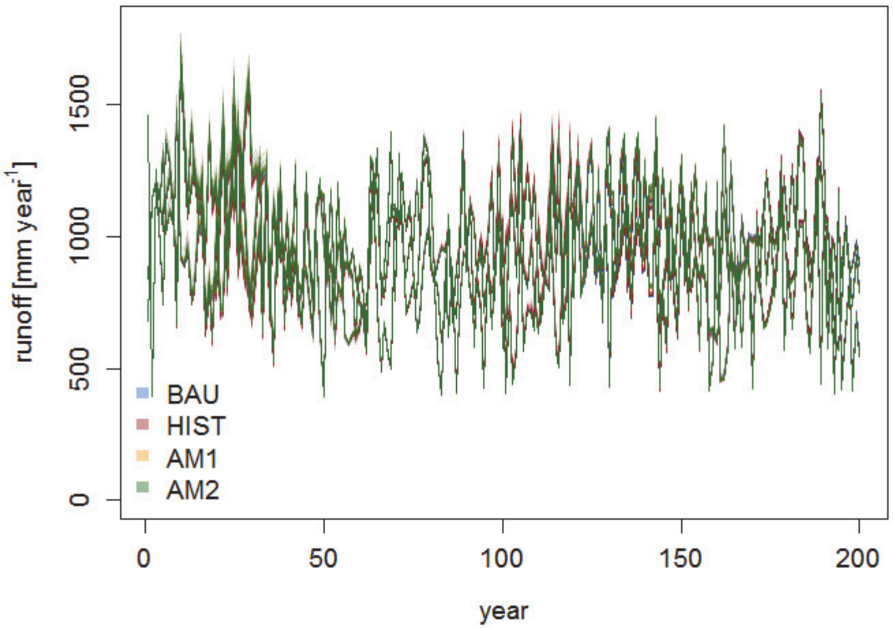

Figure S22: ES indicator protection flow (runoff) over the entire simulation period. Each line represents one run.

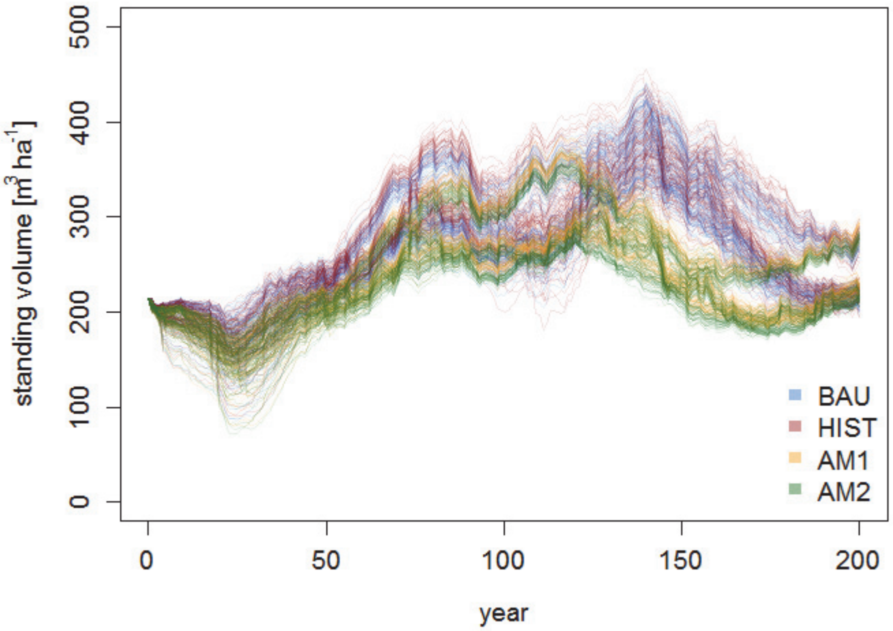

Figure S23: ES indicator timber stock (standing volume) over the entire simulation period. Each line represents one run.

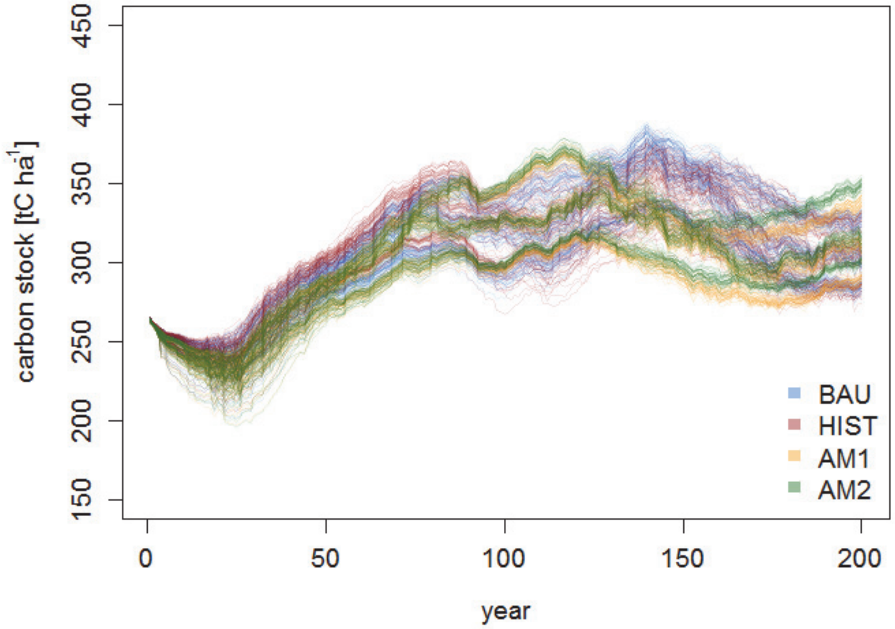

Figure S24: ES indicator carbon stock over the entire simulation period. Each line represents one run

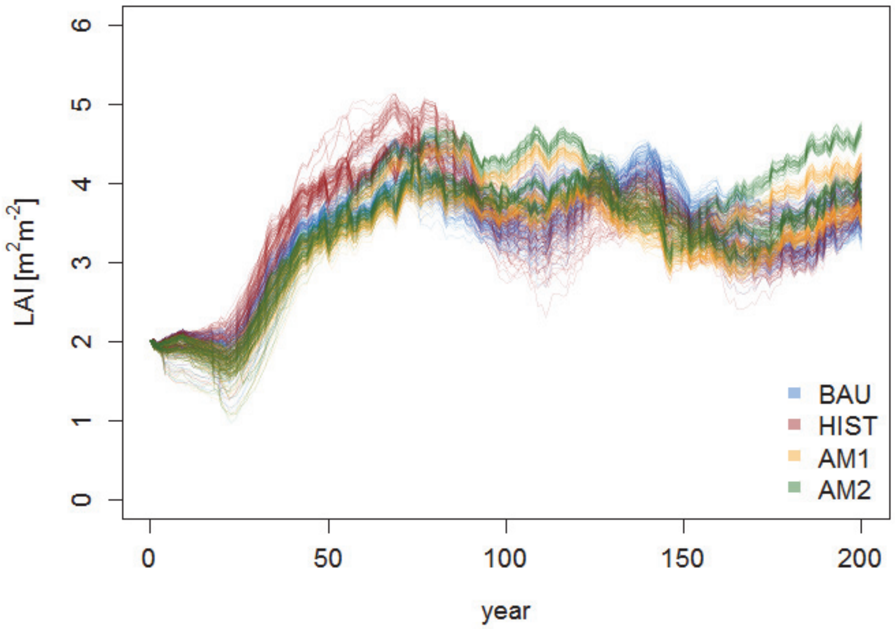

Figure S25: ES indicator protection stock (Leaf Area Index) over the entire simulation period. Each line represents one run.

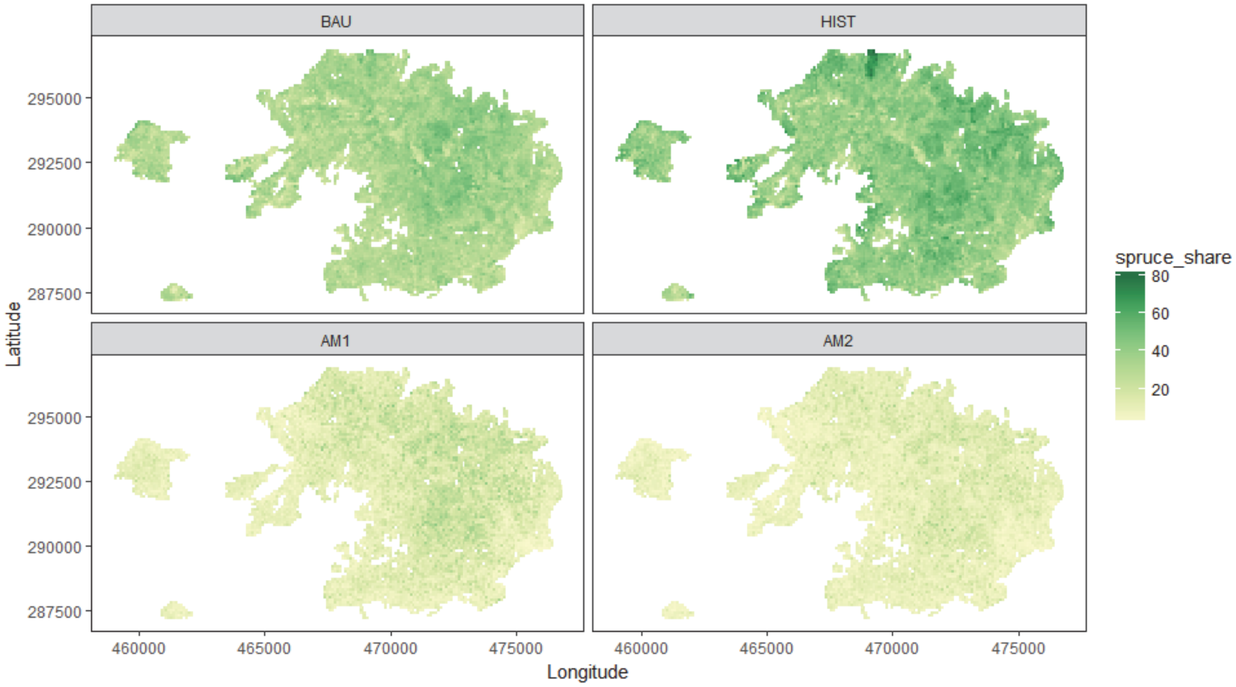

Figure S26: Spruce share for each individual 1 ha stand in the simulation year 200.

Table S4: Analysis of variance of the factors contributing to the temporal variation in ecosystem service indicator. Values are in percent of variance explained.

Indicator	Management	Climate	Disturbance	Replicate	Residual
Timber flow	36.265	45.210	0.372	8.721	9.433
Carbon flow	2.970	57.638	1.445	11.336	26.611
Protection flow	0.345	98.985	0.067	0.056	0.547
Timber stock	47.724	45.169	2.690	0.913	3.505
Carbon stock	7.093	86.945	2.693	0.844	2.424
Protection stock	12.639	69.885	7.414	1.149	8.913

Table S5: Analysis of the relationship between level and stability of ecosystem provisioning in four different time periods.

**Period**	**Level (min-max)**	**Stability (min-max)**	**Slope level~stability**	**R^2**	**P-value**	**Level (min-max)**	**Stability (min-max)**	**Slope level~stability**	**R^2**	**P-value**
	Timber flow					Timber stock				
0-200	3.583 - 6.387	0.069 - 0.149	-23.144	0.375	< 0.001	199.2 - 342.4	0.0034 - 0.0114	-15729.064	0.571	< 0.001
0-50	4.246 - 6.948	0.064 - 0.194	1.625	0.005	0.135	141.1-214.9	0.0075-0.0566	730.986	0.099	< 0.001
50-100	2.473 - 6.574	0.069 - 0.203	-24.562	0.555	< 0.001	228.1 - 363.1	0.0072 - 0.0275	-6544.842	0.655	< 0.001
100-150	1.570 - 7.506	0.045 - 0.210	-17.994	0.178	< 0.001	248.2 - 405.9	0.0066 - 0.0365	-4517.878	0.280	< 0.001
150-200	3.730 - 9.109	0.059 - 0.221	-25.964	0.377	< 0.001	182.5 - 340.7	0.0054 - 0.0577	-1706.615	0.260	< 0.001
	Carbon flow					Carbon stock				
0-200	-0.204 - 1.025	0.08 - 0.179	-0.412	0.002	0.367	281.0 - 346.2	0.006 - 0.014	-8456.64	0.674	< 0.001
0-50	-0.667 - 1.926	0.077 - 0.159	-0.275	0.000	0.825	227.6 - 263.2	0.0123 - 0.0367	-190.692	0.011	0.0231
50-100	-0.153 - 1.995	0.102 - 0.240	-2.114	0.015	0.008	288.5 - 349.2	0.0147 - 0.0582	-1555.33	0.576	< 0.001
100-150	-1.033 - 1.657	0.069 - 0.264	0.538	0.001	0.434	295.3 - 368.2	0.0152 - 0.0761	-801.708	0.271	< 0.001
150-200	-1.403 - 1.020	0.060 - 0.238	4.119	0.094	< 0.001	275.2 - 350.7	0.0134 - 0.1121	-369.337	0.136	< 0.001
	Protection flow					Protection stock				
0-200	879.9 - 1005.6	0.00113 - 0.00152	- 148066.98	0.180	< 0.001	3.278 - 4.09	0.324 -0.523	-3.0707	0.486	< 0.001
0-50	1011.2 - 1083.7	0.00108 - 0.00156	-12069.221	0.023	< 0.001	1.775 - 2.669	0.329 - 0.329	-0.39514	0.050	< 0.001
50-100	829.4 - 965.6	0.00135 - 0.00158	-391522.880	0.418	< 0.001	3.476 - 4.794	0.586 - 2.496	-0.56815	0.574	< 0.001
100-150	886.9 - 1071.2	0.00134 - 0.00160	51562.910	0.003	0.239	3.302 - 4.331	0.725 - 5.296	-0.02295	0.003	0.226
150-200	809.3 - 1008.4	0.00127 - 0.00164	-283401.630	0.247	< 0.001	2.941 - 4.279	0.828 - 4.769	0.03928	0.008	0.0525

Literature cited

Frank, G. 1992. Die natürlichen Waldsgesellschaften des Höllengebirges. Teilprojekt im Rahmen des Schutzwaldsanierungsprojektes Höllengebirge. Vienna, Austria.

Herrnegger, M., H.-P. Nachtnebel, and T. Haiden. 2012. Evapotranspiration in high alpine catchments – an important part of the water balance! Hydrology Research 43:460.

Jacob, D. 2001. A note to the simulation of the annual and inter-annual variability of the water budget over the Baltic Sea drainage basin. Meteorology and Atmospheric Physics 77:61–73.

Kilian, W., F. Müller, and F. Starlinger. 1994. Die forstlichen Wuchsgebiete Österreichs. Eine Naturraumgliederung nach Waldökologischen Gesichtspunkten. Forstliche Bundesversuchsanstalt, Vienna, Austria.

Kobler, J., R. Jandl, T. Dirnböck, M. Mirtl, and A. Schindlbacher. 2015. Effects of stand patchiness due to windthrow and bark beetle abatement measures on soil CO2 efflux and net ecosystem productivity of a managed temperate mountain forest. European Journal of Forest Research 134:683–692.

Leitgeb, E., R. Reiter, M. Englisch, P. Lüscher, P. Schad, and K.-H. Feger. 2013. Waldböden. Ein Bildatlas der wichtigsten Bodentypen aus Österreich, Deutschland und der Schweiz. First edition. Wiley-WCH, Weinheim.

Lexer, M. J., K. Hönninger, H. Scheifinger, C. Matulla, N. Groll, H. Kromp-Kolb, K. Schadauer, F. Starlinger, and M. Englisch. 2002. The sensitivity of Austrian forests to scenarios of climatic change: a large-scale risk assessment based on a modified gap model and forest inventory data. Forest Ecology and Management 162:53–72.

Nakicenovic, N., J. Alcamo, A. Grubler, K. Riahi, R. A. Roehrl, H. H. Rogner, and N. Victor. 2000. Special Report on Emissions Scenarios (SRES), A Special Report of Working Group III of the Intergovernmental Panel on Climate Change. Intergovernmental Panel on Climate Change, Cambridge University Press, Cambridge.

Neumann, M., A. Moreno, C. Thurnher, V. Mues, S. Härkönen, M. Mura, O. Bouriaud, M. Lang, G. Cardellini, A. Thivolle-Cazat, K. Bronisz, J. Merganic, I. Alberdi, R. Astrup, F. Mohren, M. Zhao, and H. Hasenauer. 2016. Creating a regional MODIS satellite-driven net primary production dataset for european forests. Remote Sensing 8:1–18.

Pal, J. S., F. Giorgi, X. Bi, N. Elguindi, F. Solmon, S. A. Rauscher, X. Gao, R. Francisco, A. Zakey, J. Winter, M. Ashfaq, F. S. Syed, L. C. Sloan, J. L. Bell, N. S. Diffenbaugh, J. Karmacharya, A. Konaré, D. Martinez, R. P. da Rocha, and A. L. Steiner. 2007. Regional Climate Modeling for the Developing World: The ICTP RegCM3 and RegCNET. Bulletin of the American Meteorological Society 88:1395–1409.

Radu, R., M. Déqué, and S. Somot. 2008. Spectral nudging in a spectral regional climate model. Tellus A: Dynamic Meteorology and Oceanography 60:898–910.

Seidl, R., W. Rammer, and M. J. Lexer. 2009. Schätzung von Bodenmerkmalen und Modellparametern für die Waldökosystemsimulation auf Basis einer Großrauminventur. Allgemeine Forst- und Jagdzeitung:35–44.

Thom, D., W. Rammer, T. Dirnböck, J. Müller, J. Kobler, K. Katzensteiner, N. Helm, and R. Seidl. 2017a. The impacts of climate change and disturbance on spatio-temporal trajectories of biodiversity in a temperate forest landscape. Journal of Applied Ecology 54:28–38.

Thom, D., W. Rammer, and R. Seidl. 2017b. Disturbances catalyze the adaptation of forest ecosystems to changing climate conditions. Global Change Biology 23:269–282.

Weinfurter, P. 2004. Waldbauhandbuch. Eine Orientierungshilfe für die Praxis. Österreichische Bundesforste AG, Ed. Purkersdorf, Austria.

Yang, W., D. Huang, B. Tan, J. C. Stroeve, N. V Shabanov, Y. Knyazikhin, R. R. Nemani, and R. B. Myneni. 2004. Analysis of Leaf Area Index and Fraction Vegetation Absorbed PAR Products from the Terra MODIS Sensor : 2000-2004. IEEE Transactions on Geoscience and Remote Sensing 44:2000–2004.

Zehetgruber, B., J. Kobler, T. Dirnböck, R. Jandl, R. Seidl, and A. Schindlbacher. 2017. Intensive ground vegetation growth mitigates the carbon loss after forest disturbance. Plant and Soil 420:239–252.
